# From fibrosis regression to recompensation: bibliometric and clinical evidence mapping of disease modification in cirrhosis

**DOI:** 10.3389/fmed.2026.1921542

**Published:** 2026-08-20

**Authors:** Han-Wen Zhang, Jun-Ling Wu, Yi Chen, Zhi-Zhuang Zhao, Shu-Huang Peng, Jing-Xuan Zhao, Xian-Ya Huang, Zhe Luan, Shu-Fang Wang, Jiang-Tao Liu, Gang Sun

**Affiliations:** 1 Medical School of Chinese PLA, Beijing, China; 2 Department of Gastroenterology and Hepatology, The First Medical Center, Chinese PLA General Hospital, Beijing, China; 3 Department of Geriatrics, Hainan Hospital of Chinese PLA General Hospital, Sanya, China; 4 Department of Gastroenterology and Hepatology, Hainan Hospital of Chinese PLA General Hospital, Sanya, China

**Keywords:** bibliometric analysis, cirrhosis, CiteSpace, decompensated cirrhosis, disease modification, fibrosis regression, recompensation

## Abstract

**Background:**

The Baveno VII consensus formally defined cirrhosis recompensation in 2022, marking an important milestone in the transition of cirrhosis management from complication control toward disease modification. This concept did not emerge abruptly but was built on decades of research concerning etiological control, fibrosis regression, portal hypertension control, liver function recovery, and reversal of decompensation. However, the evolution of these knowledge domains, the key milestones shaping the field, and the clinical or mechanistic directions that should be prioritized remain unclear. This study used bibliometric analysis to map the development and trends of cirrhosis recompensation-related research, with emphasis on recent advances and future directions.

**Methods:**

Relevant publications (up to May 31, 2026) were retrieved from the Web of Science Core Collection (WoSCC) and PubMed. Bibliometric visualization and network analyses were performed using CiteSpace, VOSviewer, bibliometrix, R, and Python to examine publication trends, global collaborations, core journals, author networks, co-cited references, keyword evolution, citation bursts, and randomized controlled trial (RCT) evidence mapping.

**Results:**

A total of 588 WoSCC publications and 168 PubMed RCT publications were included. The field showed a three-stage evolution: complication control and survival prolongation; etiology-directed therapy and fibrosis regression; and recompensation-centered disease modification after Baveno VII. The United States and China were leading contributors, while Journal of Hepatology and Hepatology were central journals. Co-citation and keyword analyses highlighted antiviral therapy, fibrosis regression, portal hypertension, and recompensation as major knowledge themes. Further within-WoSCC mapping of the 31-publication RCT subset showed that no publication evaluated formal Baveno VII recompensation as an explicit endpoint. Complementary analysis of the 168 PubMed RCT publications showed that recompensation was not evaluated as an endpoint even among publications reporting follow-up beyond 48 weeks, indicating that this gap could not be attributed solely to short follow-up.

**Conclusion:**

This bibliometric analysis delineates the evolving landscape of cirrhosis recompensation-related research, characterized by a transition from complication control to disease modification and an increasing focus on recompensation after Baveno VII. Persistent gaps in RCT evidence, especially regarding recompensation endpoints and long-term disease-modification outcomes, underscore the need for continued high-quality clinical and translational studies in this field.

## 1 Introduction

Cirrhosis represents the end stage of chronic liver injury and remains a major cause of liver-related morbidity and mortality worldwide (1). Global estimates indicate that cirrhosis causes more than one million deaths each year and accounted for approximately 41.4 million disability-adjusted life years (DALYs) in 2017 (2). Once decompensation occurs, patients face a substantially increased risk of recurrent hospitalization, liver transplantation, and death (3, 4). Conventional management of decompensated cirrhosis has therefore focused primarily on controlling complications, including ascites, variceal bleeding, hepatic encephalopathy, infection, and hepatorenal syndrome (HRS), while improving short-term stability and prolonging survival. Although this complication-centered approach has improved clinical outcomes, it does not fully address whether the underlying disease course can be modified or whether a decompensated patient can return to a more stable clinical state.

Historically, cirrhosis was often regarded as an irreversible or only minimally reversible condition (5, 6). This view has been progressively challenged by advances in etiology-directed therapy, especially antiviral treatment for chronic hepatitis B and hepatitis C, as well as by improved methods for monitoring fibrosis and liver function over time. The 2010 entecavir study by Chang et al. provided important histological evidence that advanced fibrosis and cirrhosis could regress during long-term viral suppression, marking a key transition from viewing cirrhosis as a fixed end-stage condition toward considering it a potentially modifiable disease state (7). Subsequent studies on sustained virological response, fibrosis regression, non-invasive fibrosis assessment, and liver function recovery further strengthened this disease-modification concept (8–10).

However, fibrosis regression alone does not fully capture the clinical recovery of patients with decompensated cirrhosis. The Baveno VII consensus advanced the field by formally defining recompensation as an integrated clinical state rather than a single histological or biochemical improvement (11). According to this concept, recompensation requires sustained control or removal of the primary etiological factor, resolution of major decompensating events over time, and improvement in liver function. This definition connects etiological control, portal hypertension-related events, and hepatic functional recovery within a unified clinical endpoint, thereby shifting the research focus from structural regression alone toward patient-level reversal of decompensation.

But the emergence of recompensation in recent years also creates a challenge for understanding the field. Because this term was standardized only in 2022, much of the relevant historical literature did not use recompensation explicitly, but was distributed across related topics such as cirrhosis regression, decompensation reversal, symptom resolution, transplant delisting, and liver function recovery. As a result, the development of this research area cannot be adequately captured by a narrow term-based review. Bibliometric analysis is therefore well suited to this topic because it can systematically map landmark references, co-citation structures, keyword evolution, and emerging hotspots, thereby reconstructing how disease-modification research in cirrhosis has evolved toward the current concept of recompensation and emerging research hotspots (12–15).

Nevertheless, bibliometric attention does not necessarily indicate mature clinical evidence. A topic may become highly visible in publications and citations before being incorporated into randomized controlled trial (RCT) design or standardized clinical endpoints (15). This issue is particularly relevant for recompensation, which has rapidly gained conceptual importance after Baveno VII but may not yet be widely adopted as an explicit endpoint in interventional trials. Therefore, the WoSCC dataset was used for bibliometric analysis, and an internally derived RCT subset was used for within-dataset comparison between major research hotspots and clinical trial endpoints. The PubMed RCT dataset was analyzed separately to provide complementary evidence on keyword patterns, endpoint selection, follow-up durations and other characteristics. We aimed to characterize the global research landscape, knowledge structure, and thematic evolution from fibrosis regression to formal recompensation, and to identify gaps between major research themes and available interventional evidence.

## 2 Materials and methods

### 2.1 Data sources and search strategy

This study combined bibliometric analysis with complementary mapping of RCT evidence. WoSCC was searched up to May 31, 2026 and used for the bibliometric analysis. An internally derived RCT subset was extracted from the same dataset to compare major bibliometric hotspots with the clinical trial endpoints evaluated in the included publications. PubMed was searched separately over the same period, and the resulting RCT dataset was used for complementary descriptive analyses of keyword patterns, endpoint selection, and follow-up durations. Considering that cirrhosis recompensation was formally proposed as a new concept in the Baveno VII consensus in 2022, a multi-step search strategy was used to ensure comprehensive literature retrieval. Five thematic search sets were constructed and combined, covering recompensation, decompensation reversal, fibrosis/cirrhosis regression, symptom resolution, and liver function recovery (11). The complete WoSCC and PubMed search strategies are provided in Supplementary Table 1.

### 2.2 Inclusion and exclusion criteria

Bibliographic records retrieved from the WoSCC and PubMed were exported and processed separately using EndNote 21. For the WoSCC bibliometric dataset, eligible records were limited to English-language original articles and reviews to ensure consistent text processing across software platforms and the feasibility of manual screening. Potential duplicates were checked according to digital object identifiers, titles, publication years, and author information, followed by manual verification when necessary. Publications were excluded if they did not meet the specified document-type, language, subject-category, or topic-relevance criteria. Within the final WoSCC dataset, RCT publications were manually identified to construct the internally derived WoSCC RCT subset.

For the PubMed RCT dataset used for complementary analyses, eligibility was restricted to English-language publications reporting randomized controlled trials involving patients with cirrhosis or portal hypertension. These publications were screened for relevance to cirrhosis disease modification, regression or reversal, liver function recovery, portal hypertension control, and decompensation-related outcomes. Eligible publications were required to evaluate therapeutic interventions and report clinically relevant outcomes within at least one of these domains. Non-randomized controlled clinical trials were not eligible. Publications were excluded if they did not involve a relevant population or therapeutic intervention or were unrelated to the target topic after screening.

Two reviewers independently assessed titles and abstracts, and disagreements were resolved through discussion with a third reviewer. Full texts were reviewed when eligibility could not be determined from titles and abstracts. Records lacking essential bibliographic information were excluded. Before analysis, author names, institutional affiliations, journal titles, and keywords were standardized across the final datasets. Detailed search strategies and screening procedures are provided in Supplementary Table 1.

### 2.3 Bibliometric analysis and visualization

Bibliometric visualization and network analyses were performed using CiteSpace 7.0, VOSviewer 1.6.21, R 4.4.2, bibliometrix 5.4.1, and Python 3.13.2 (12–14). CiteSpace was used for collaboration networks, co-citation analysis, keyword co-occurrence, clustering, timeline visualization, and burst detection; bibliometrix was used for descriptive bibliometric indicators and thematic mapping; and VOSviewer was used to generate the journal citation network shown in Figure 4C. For CiteSpace analyses, the time span was set according to each dataset period, with 1 year per slice. Node types were analysed separately, including country/region, institution, author, co-cited author, co-cited reference, and keyword. Country/region, institution, and author productivity and collaboration analyses used full counting. Each publication was credited in full to every country/region and institution represented in the affiliations and to every listed author; consequently, entity-level publication counts are not additive. Citation counts were taken as indexed in WoSCC and included self-citations, because no self-citation exclusion was applied. The selection criterion was set to the g-index with *k* = 25, and Pathfinder, pruning sliced networks, and pruning the merged network were applied. For the within-WoSCC evidence mapping, rule-based matching applied the same non-exclusive clinical theme dictionary (Supplementary Table 8) to titles, abstracts, author keywords, and Keywords Plus in the full dataset and RCT subset, with ambiguous assignments verified at the record level; PubMed matching used titles, abstracts, and available keyword fields (Supplementary Table 8). The evidence-gap matrix compared the prevalence of major bibliometric themes in the full dataset with the representation of the corresponding clinical endpoints in the RCT subset. The intervention–outcome Sankey map summarized publication-level links using full link counting. Because the themes were non-exclusive, one publication could contribute to multiple intervention–outcome links. The PubMed RCT dataset was analyzed separately using descriptive methods to characterize keyword patterns, clinical endpoints, follow-up durations, and other trial characteristics. All evidence-mapping visualizations were generated using Python, pandas 2.2.3, and Matplotlib 3.10.5.

## 3 Results

**FIGURE 1:**
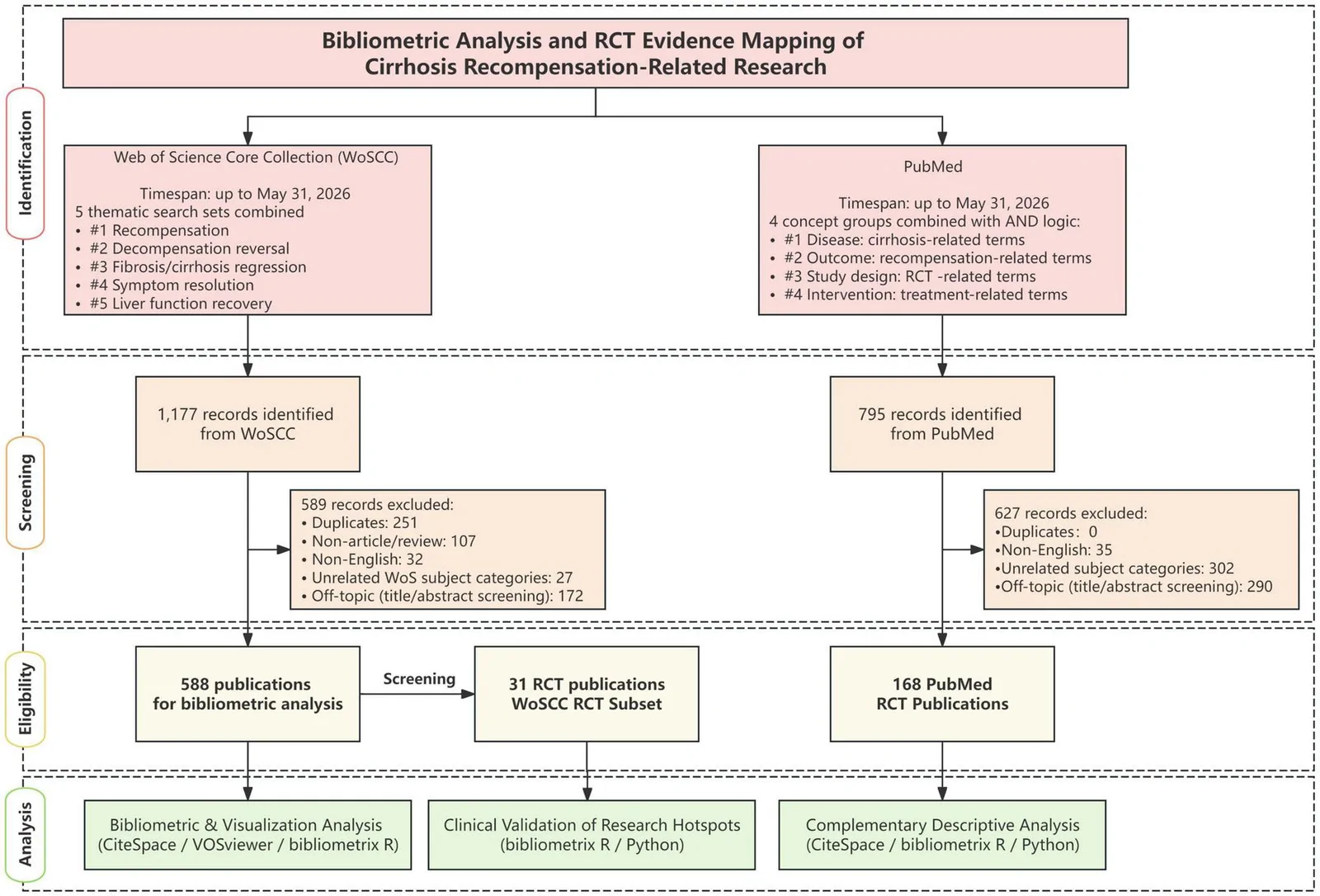
Flow diagram of literature retrieval, screening, dataset construction, bibliometric analysis, and clinical evidence mapping.

The WoSCC search identified 1,177 records, of which 588 publications were retained for bibliometric analysis. Among these, 31 publications were included in the internally derived WoSCC RCT subset. The PubMed search identified 795 records, of which 168 RCT publications met the eligibility criteria for complementary descriptive analysis (Figure 1).

### 3.1 Temporal publication trends

**FIGURE 2:**
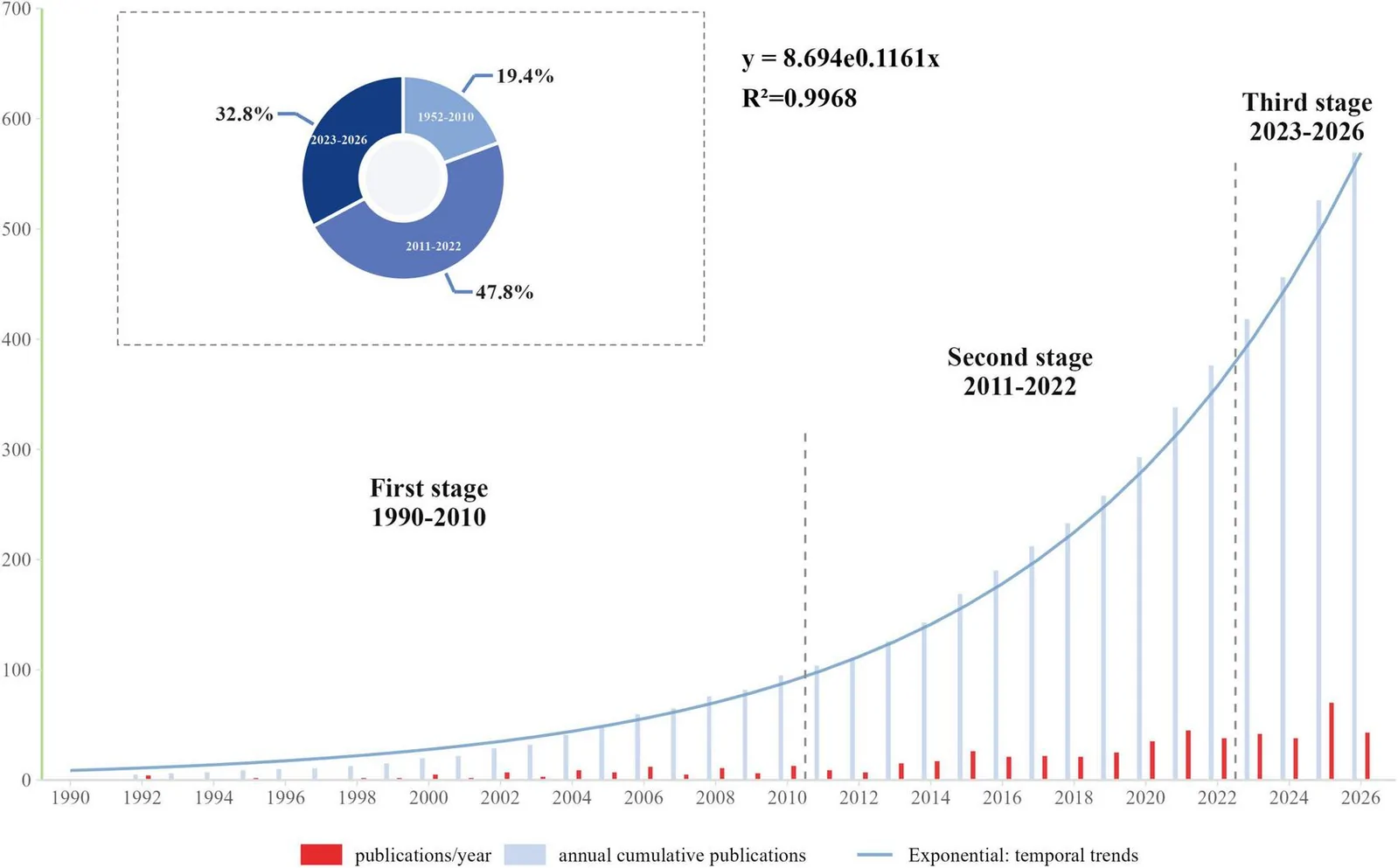
Annual publication trend and stage-sensitive evolution of cirrhosis disease-modification and recompensation-related research. Red bars represent annual publication counts, pale-blue bars represent cumulative publications, and the solid curve represents the fitted exponential trend. The inset shows the proportion of the 588 WoSCC publications assigned to each stage. Dashed lines indicate the prespecified 2010 and 2022 transition points. Publications from 2026 were retrieved only up to May 31 and therefore represent an incomplete year.

The annual publication trend demonstrated sustained growth in cirrhosis regression research from 1990 to 2026 (Figure 2). Based on the developmental trajectory of the field and two landmark citation events, a three-stage temporal classification was constructed (Figure 2). In the first stage (through 2010), research output remained limited and grew slowly. Following the publication of the landmark entecavir study by Chang et al. in 2010, which fundamentally challenged the long-held view of cirrhosis irreversibility by providing histological evidence of cirrhosis reversal during long-term antiviral therapy (7), the field entered the second stage (2011–2022). During this period, cirrhosis reversal gained increasing recognition as a viable research paradigm, with annual output rising steadily and accounting for 47.8% of total publications. The publication of the Baveno VII consensus in 2022, which formally defined recompensation as a clinically meaningful endpoint (11), marked the onset of the third stage (2023–2026). This most recent period witnessed a rapid acceleration in research activity, with 32.8% of all publications (*n* = 193) concentrated within only 4 years. To quantitatively assess the proposed stage boundaries, publication rates were compared across the three prespecified periods. The annual rate increased from 1.9 publications per year up to 2010 (*n* = 114) to 23.4 per year during 2011–2022 (*n* = 281), corresponding to a rate ratio of 12.12 (95% CI, 9.75–15.07; *P* < 0.001). It further increased to 50.0 publications per year during 2023–2025 (*n* = 150), with a rate ratio of 2.14 relative to 2011–2022 (95% CI, 1.75–2.60; *P* < 0.001). Consistently, the fitted cumulative publication slopes increased from 1.4 to 25.8 and 56.7 publications per year across the three stages. These findings quantitatively supported marked accelerations in research activity across the prespecified 2010 and 2022 transition points (Supplementary Figure 3 and Supplementary Table 2). Overall, the cumulative publication curve followed an exponential growth model (R^2^ = 0.9968).

### 3.2 Geographical and institutional contributions

**FIGURE 3:**
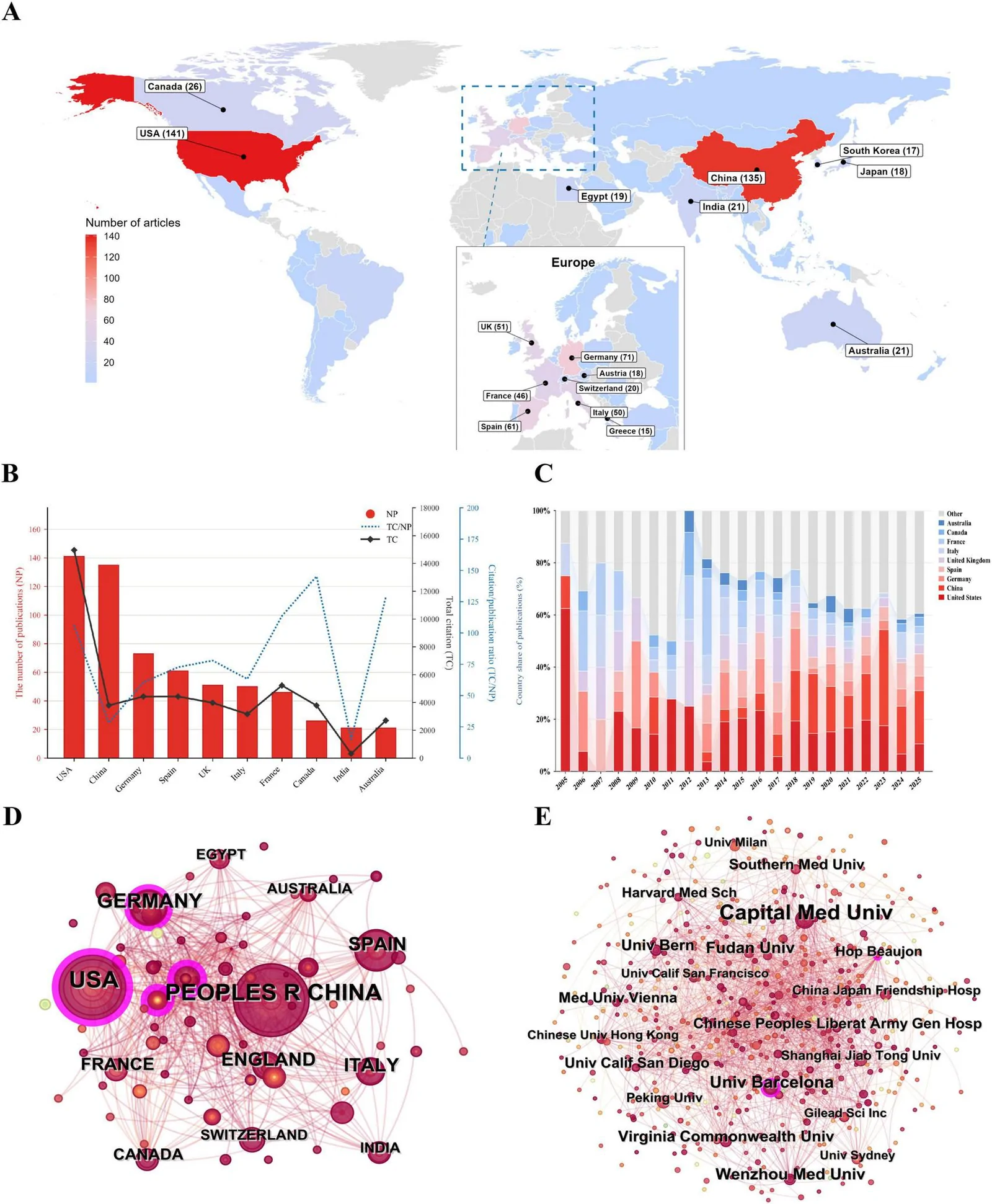
Global geographical and institutional contributions. Panels show country/region productivity, citation impact, annual country distribution, international collaboration, and institutional collaboration networks. **(A)** Country/region color intensity represents the number of publications under full counting. **(B)** Red bars indicate the number of publications, the black line indicates total citations, and the blue dotted line indicates citations per publication. **(C)** Annual shares were calculated from full-counted country/region contributions, with the remaining countries grouped as Other. **(D,E)** Node size represents publication output, links represent co-authorship, and link width reflects collaboration frequency. Time-coded node and link colors range from earlier to later years, and purple outer rings indicate betweenness centrality of at least 0.10. Country, region, and institution counts were based on author affiliations; each publication contributed one count to every represented entity and counts are therefore not additive. CiteSpace networks were selected using the g-index with *k* = 25 in one-year slices and pruned using Pathfinder, pruning sliced networks, and pruning the merged network. The denominator was 588 WoSCC publications.

A total of 74 countries and regions contributed to the 588 included publications. The United States ranked first with 141 articles, followed by China with 135, Germany with 71, and Spain with 61 (Figure 3A; Supplementary Table 3). In terms of citation impact, the United States also had the highest total citations (TC = 14,956; TC/NP = 106.07), followed by France (TC = 5,220; TC/NP = 113.48), Spain (TC = 4,426; TC/NP = 72.56), Germany (TC = 4,379; TC/NP = 61.68), and China (TC = 3,789; TC/NP = 28.07) (Figure 3B and Table 1). The annual country distribution showed that the United States contributed substantially in the earlier period, whereas China’s publication share increased in recent years (Figure 3C). The thematic profiles of China and the United States also differed. China-linked publications showed greater representation of HBV-related research (61.5% vs. 33.3%), recompensation or decompensation reversal (31.1% vs. 17.0%), and traditional Chinese medicine (8.1% vs. 2.1%). In contrast, United States-linked publications more frequently addressed HCV-related research (23.4% vs. 8.1%) and metabolic or steatotic liver disease (26.2% vs. 10.4%). These differences remained significant after excluding bilateral publications and adjusting for multiple comparisons. Fibrosis or cirrhosis regression was prominent in both countries, while portal-hypertension-related research showed no clear difference (Supplementary Table 4).

**FIGURE 4:**
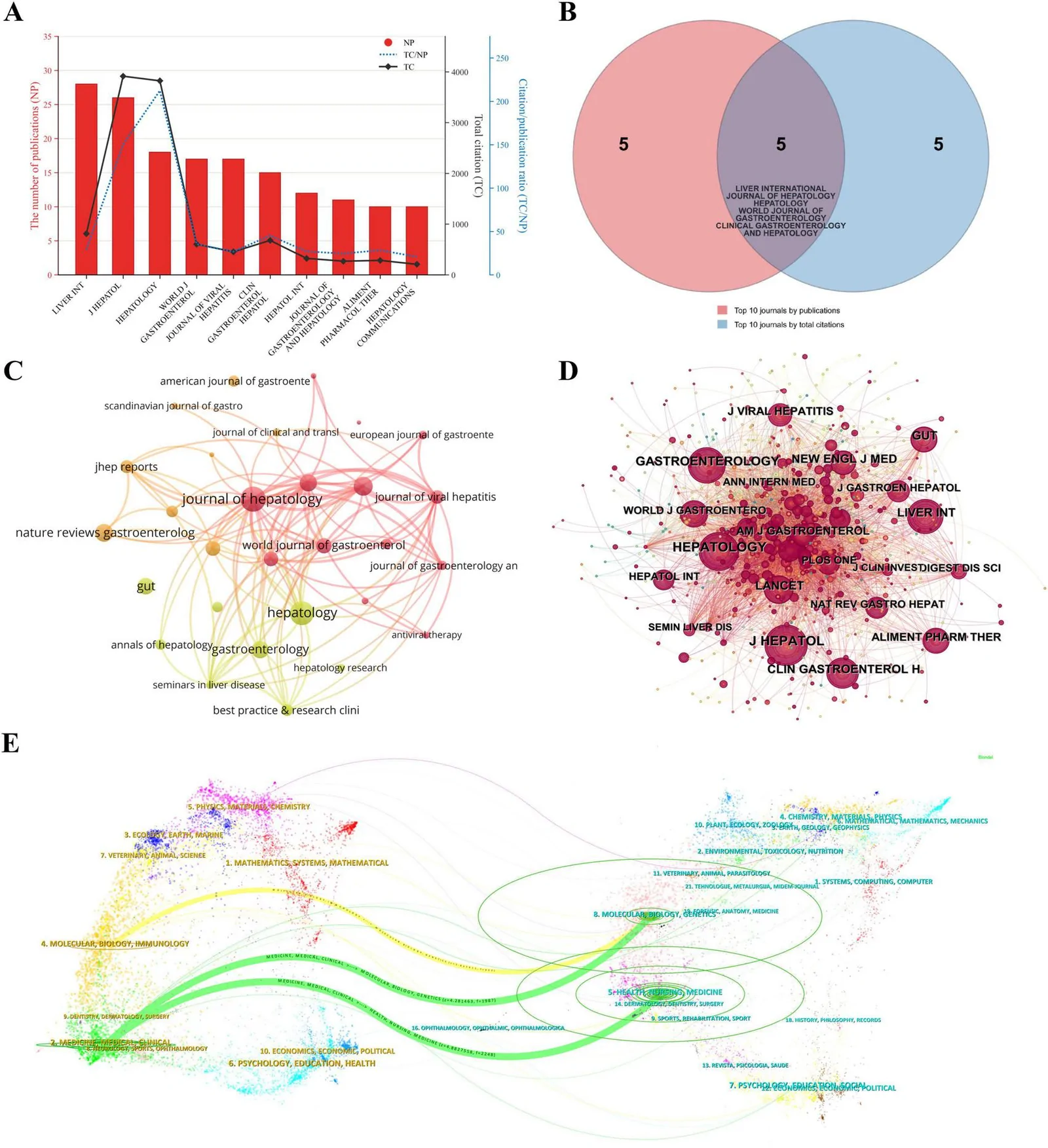
Journal distribution and interdisciplinary knowledge flow. Panels summarize publication and citation patterns, journal citation networks, co-cited journals, and the dual-map overlay. **(A)** Red bars represent the number of publications, the black line represents total citations, and the blue dotted line represents citations per publication. **(B)** The Venn diagram shows overlap between the ten most productive and ten most cited journals. **(C)** In the VOSviewer journal citation network, node size represents journal publication output, links represent direct citation relationships, link width reflects total link strength, and colors identify journal clusters without ordinal meaning. **(D)** In the CiteSpace co-cited-journal network, node size represents co-citation frequency, links indicate that two journals were cited together, and link width reflects co-citation strength. Time-coded colors indicate the year of co-citation, and purple rings indicate betweenness centrality of at least 0.10. The network used the g-index with *k* = 25 and the stated pruning procedures. **(E)** The dual-map overlay displays citing journal domains on the left and cited domains on the right; colored trajectories indicate major citation pathways. Analyses were based on 588 WoSCC publications.

**TABLE 1:** Top 10 countries/regions ranked by research volume.

Rank	Country/Region	Number of publications (NP)	Total citations (TC)	TC/NP
1	United States	141	14956	106.07
2	China	135	3789	28.07
3	Germany	71	4379	61.68
4	Spain	61	4426	72.56
5	United Kingdom	51	3974	77.92
6	Italy	50	3158	63.16
7	France	46	5220	113.48
8	Canada	26	3776	145.23
9	Australia	21	2703	128.71
10	India	21	312	14.86

International collaboration was identified in 139 multi-country publications, while 449 publications were produced within a single country or region. The collaboration network showed dense links among the United States, China, Germany, Spain, France, Italy, and the United Kingdom (Figure 3D). The strongest bilateral collaboration was observed between China and the United States, followed by collaborations between the United States and Canada, Spain, Germany, and the United Kingdom.

At the institutional level, the top ten institutions contributed 180 publications in total (Table 2). Capital Medical University ranked first with 40 publications, followed by the University of Barcelona with 22 publications, the University of California San Diego with 17 publications, and Wenzhou Medical University and Fudan University with 16 publications each. The University of California San Diego had the highest citation count among the top ten institutions (TC = 2,719; TC/NP = 159.94), followed by Virginia Commonwealth University (TC = 1,253; TC/NP = 96.38) and Harvard Medical School (TC = 1,028; TC/NP = 79.08). The institutional collaboration network was mainly centered on Capital Medical University, Fudan University, Southern Medical University, the University of Barcelona, and the University of California San Diego (Figure 3E).

**TABLE 2:** Top 10 institutions ranked by research volume.

Rank	Institution	Affiliation	Number of publications (NP)	Total citations (TC)	TC/NP
1	Capital Medical University	China	40	1012	25.3
2	University of Barcelona	Spain	22	943	42.86
3	University of California San Diego	United States	17	2719	159.94
4	Wenzhou Medical University	China	16	469	29.31
5	Fudan University	China	16	334	20.88
6	Southern Medical University	China	15	338	22.53
7	University of Bern	Switzerland	15	320	21.33
8	Virginia Commonwealth University	United States	13	1253	96.38
9	Harvard Medical School	United States	13	1028	79.08
10	Medical University of Vienna	Austria	13	535	41.15

NP, number of publications; TC, total citations; TC/NP, average citations per publication. Country/region and institution publication counts were calculated using full counting and are therefore not additive across entities. TC values include self-citations as indexed in WoSCC.

### 3.3 Journal distribution and interdisciplinary knowledge flow

A total of 249 journals contributed to the 588 included publications. Liver International had the largest publication output (NP = 28), followed by Journal of Hepatology (NP = 26) and Hepatology (NP = 18). However, the citation distribution showed a different pattern. Journal of Hepatology and Hepatology ranked first and second in total citations, with 3,914 and 3,823 citations, respectively, and also had high TC/NP ratios (150.54 and 212.39) (Figure 4A and Table 3). These findings indicate that the two hepatology-focused journals served as both major publication venues and highly cited sources in this field. In addition, several journals with fewer publications showed high citation efficiency, particularly Lancet (NP = 2; TC/NP = 1,605.50), Gastroenterology (NP = 7; TC/NP = 473.43), New England Journal of Medicine (NP = 2; TC/NP = 347.00), and Annals of Internal Medicine (NP = 3; TC/NP = 313.33) (Table 3). The Venn diagram showed that five journals overlapped between the top ten journals by publication volume and the top ten journals by total citations: Liver International, Journal of Hepatology, Hepatology, World Journal of Gastroenterology, and Clinical Gastroenterology and Hepatology (Figure 4B).

**TABLE 3:** Leading and highly cited journals in cirrhosis disease-modification and recompensation-related research.

Journals	Number of publications (NP)	Total citations (TC)	TC/NP	Publisher country/region	2025 IF	2025 JIF quartile
Journal of Hepatology	26	3914	150.54	Netherlands	40.1	Q1
Hepatology	18	3823	212.39	United States	18	Q1
Gastroenterology	7	3314	473.43	United States	29.7	Q1
Lancet	2	3211	1605.50	United States	109	Q1
Annals of Internal Medicine	3	940	313.33	United States	17.2	Q1
Liver International	28	810	28.93	United States	6.2	Q1
New England Journal of Medicine	2	694	347.00	United States	84.5	Q1
Clinical Gastroenterology and Hepatology	15	679	45.27	United States	16.2	Q1
Gut	6	626	104.33	England	24.6	Q1
World Journal of Gastroenterology	17	603	35.47	United States	7.7	Q1

NP, number of publications; TC, total citations; TC/NP, average citations per publication; IF, impact factor; JIF, journal impact factor. JIFs and quartiles were obtained from the 2026 edition of Clarivate Journal Citation Reports and refer to 2025 values. Publisher country/region denotes the publisher location recorded in the journal profile.

The journal network further showed that Journal of Hepatology occupied a central position and was closely connected with Hepatology, Gastroenterology, World Journal of Gastroenterology, and Nature Reviews Gastroenterology and Hepatology (Figure 4C). In the co-cited journal network, Journal of Hepatology, Hepatology, Gastroenterology, Liver International, Gut, Lancet, New England Journal of Medicine, Clinical Gastroenterology and Hepatology, and World Journal of Gastroenterology appeared as major nodes (Figure 4D).

The dual-map overlay demonstrated the interdisciplinary citation pathways between citing and cited journal domains (Figure 4E). The main citation trajectories originated predominantly from Medicine/Medical/Clinical and Molecular/Biology/Immunology journals and were directed mainly toward Health/Nursing/Medicine and Molecular/Biology/Genetics journals. These patterns indicate that cirrhosis recompensation research is primarily published in clinical hepatology and medical journals, while its cited knowledge base extends to molecular biology, genetics, and broader biomedical fields. This citation structure suggests an active clinical research landscape that continues to integrate mechanistic and translational evidence.

### 3.4 Author collaboration and co-cited authors

A total of 3,957 authors contributed to the included publications. The author collaboration network showed a dense but clustered structure, with several high-productivity groups centered around major liver disease research institutions (Supplementary Figure 1A). Jidong Jia and Hong You ranked first in publication output, with 20 articles each, followed by Yameng Sun (*n* = 14), Rohit Loomba (*n* = 12), Yongping Yang (*n* = 12), Xiaojuan Ou (*n* = 12), and Xiaoning Wu (*n* = 12) (Table 4). Authors from Capital Medical University formed the largest domestic collaborative cluster, represented by Jidong Jia, Hong You, Yameng Sun, Xiaojuan Ou, and Xiaoning Wu. The Chinese People’s Liberation Army General Hospital also contributed an important collaborative group, including Yongping Yang and Yan Chen. Internationally, Rohit Loomba, Arun J. Sanyal, Thomas Reiberger, Patrick Marcellin, and Virginia Hernandez-Gea represented major overseas contributors.

**TABLE 4:** Top 10 prolific authors.

Rank	Authors	Counts	Institution
1	Jidong Jia	20	Capital Medical University
2	Hong You	20	Capital Medical University
3	Yameng Sun	14	Capital Medical University
4	Rohit Loomba	12	University of California San Diego
5	Yongping Yang	12	Chinese People’s Liberation Army General Hospital
6	Xiaojuan Ou	12	Capital Medical University
7	Xiaoning Wu	12	Capital Medical University
8	Arun J Sanyal	11	Virginia Commonwealth University
9	Jialing Zhou	10	Capital Medical University
10	Thomas Reiberger	9	Medical University of Vienna
TOTAL	Top 10 total	132	/

Author publication counts represent the number of WoSCC publications authored by each author and were calculated using full counting, with each co-author receiving one full publication credit.

The co-cited author network reflected the intellectual base of cirrhosis recompensation-related research (Supplementary Figure 1B). The most frequently co-cited authors were Marcellin P (*n* = 146), Poynard T (*n* = 129), Bedossa P (*n* = 104), Friedman SL (*n* = 102), de Franchis R (*n* = 99), Castéra L (*n* = 96), the European Association for the Study of the Liver (*n* = 91), Wanless IR (*n* = 84), Chang TT (*n* = 82), and Rockey DC (*n* = 75) (Table 5). These highly co-cited nodes were mainly associated with antiviral therapy, fibrosis assessment, portal hypertension, histological evaluation, and international consensus development. Overall, the result indicates that the field is supported by both active Chinese clinical research groups and an international knowledge base derived from fibrosis biology, antiviral treatment, and portal hypertension research.

**TABLE 5:** Top 10 co-cited authors.

Rank	Co-cited authors	Citations	Institution
1	Marcellin P	146	Hôpital Beaujon, AP-HP / Université Paris Cité, France
2	Poynard T	129	Pitié-Salpêtrière Hospital, AP-HP / Sorbonne University, France
3	Bedossa P	104	Hôpital Beaujon, AP-HP / Université Paris Cité, France
4	Friedman SL	102	Icahn School of Medicine at Mount Sinai, United States
5	de Franchis R	99	University of Milan, Italy
6	Castéra L	96	Hôpital Beaujon, AP-HP / Université Paris Cité, France
7	European Assoc Study Liver	91	European Association for the Study of the Liver, Switzerland
8	Wanless IR	84	University of Toronto, Canada
9	Chang TT	82	National Cheng Kung University Hospital, Taiwan
10	Rockey DC	75	Medical University of South Carolina, United States
TOTAL	Top 10 total	1008	/

Citation counts represent co-citation frequencies in the co-cited author analysis rather than the total citations received by each author’s publications.

### 3.5 Co-cited references and landmark citation events

**FIGURE 5:**
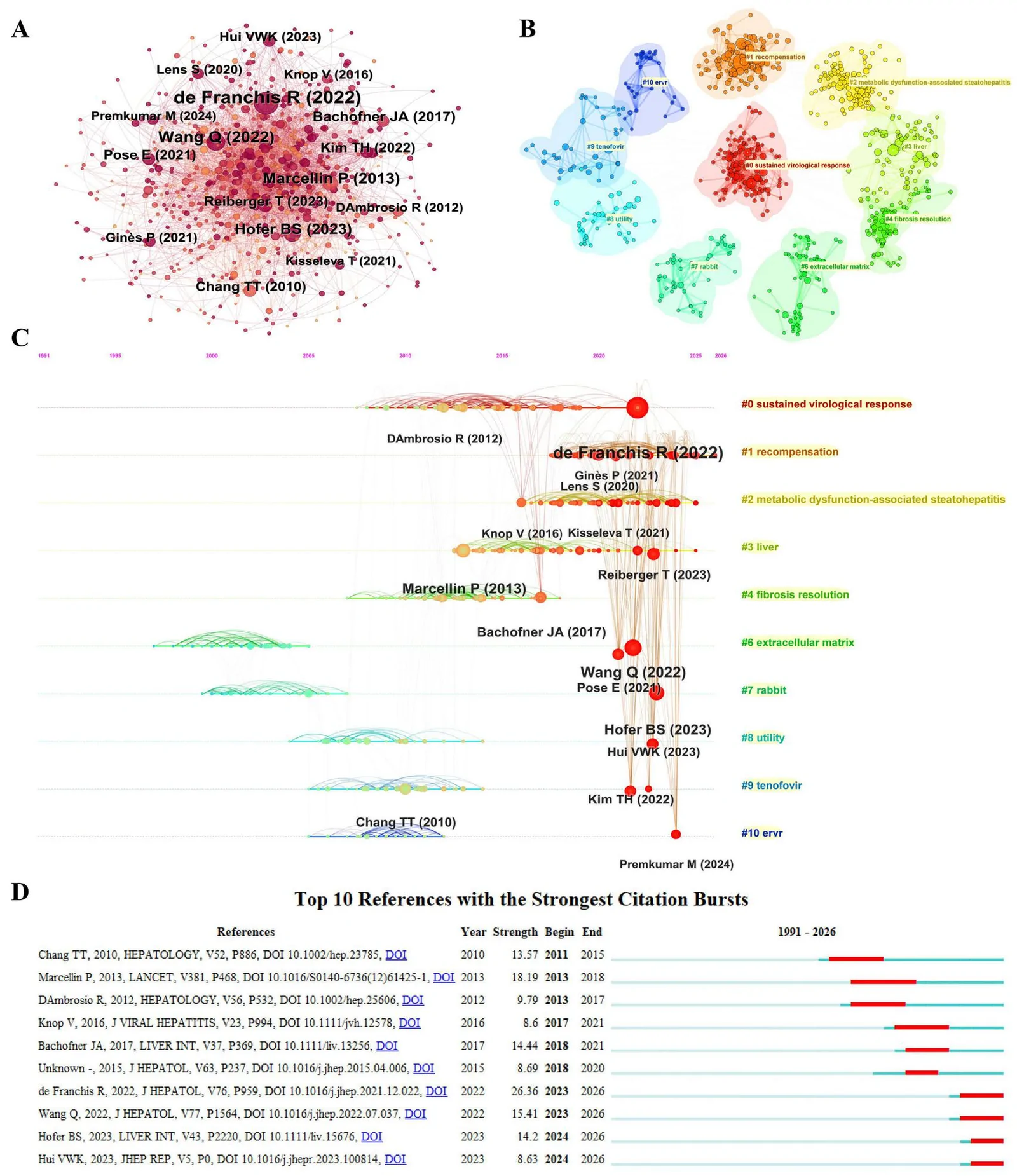
Co-cited reference analysis and landmark citation events. Panels show the reference co-citation network, clusters, timeline visualization, and references with the strongest citation bursts. **(A)** Node size represents co-citation frequency, links indicate that two references were cited together, and link width reflects co-citation strength. Time-coded colors indicate the year of co-citation, and purple outer rings indicate betweenness centrality of at least 0.10. **(B)** Colors identify reference clusters labelled using the log-likelihood ratio algorithm; cluster #0 is the largest cluster. **(C)** Nodes are arranged by publication year within each cluster, with node size representing co-citation frequency and links representing co-citation relationships. **(D)** Red segments indicate periods of statistically detected citation bursts, whereas the remaining timeline indicates the observation period. Strength, beginning year, and ending year are shown for each burst. Networks were generated from the 588 WoSCC publications using the g-index with *k* = 25 and the stated pruning procedures.

The co-cited reference analysis further supported the three-stage evolution described in the temporal publication analysis. The co-citation network contained 1,315 reference nodes (Figure 5A). The Baveno VII consensus by de Franchis et al. (11) was the most frequently co-cited reference (*n* = 84), followed by Wang Q 2022 (*n* = 45) (16), Marcellin P 2013 (*n* = 38) (8), Hofer BS 2023 (*n* = 36) (17), Bachofner JA 2017 (*n* = 28) (18), Reiberger T 2023 (*n* = 24) (19), and Chang TT 2010 (*n* = 24) (7). These references were mainly related to antiviral therapy, fibrosis or cirrhosis regression, portal hypertension, clinical outcomes, and recompensation (Supplementary Table 5).

Reference co-citation clustering identified major knowledge domains, including sustained virological response, recompensation, metabolic dysfunction-associated steatohepatitis, fibrosis resolution, extracellular matrix, tenofovir, and utility assessment (Figure 5B). The timeline visualization showed that Chang TT 2010 was located at the beginning of the antiviral-response and fibrosis-regression knowledge base, corresponding to the transition from the first to the second stage (Figure 5C) (7). Notably, no earlier reference appeared among the strongest citation bursts, supporting its role as the first major citation event in this field. Subsequent references, including D’Ambrosio R 2012 (10), Marcellin P 2013 (8), Knop V 2016 (20), and Bachofner JA 2017 (18), reflected the consolidation of etiology-directed cirrhosis regression research.

In contrast, de Franchis was positioned at the center of the recent recompensation-focused period and represented the defining citation event of the third stage. Citation-burst analysis showed that Chang TT 2010 had an early burst from 2011 to 2015 (strength = 13.57), whereas de Franchis showed the strongest and most recent burst from 2023 to 2026 (strength = 26.36) (Figure 5D) (7, 11). Recent burst references, including Wang Q 2022 (16), Hofer BS 2023 (17), Hui VWK 2023 (21), and Reiberger T 2023 (19), emerged after Baveno VII and were closely aligned with recompensation-oriented research, indicating that the Baveno VII definition has become the central reference point for current studies.

### 3.6 Keyword analysis

**FIGURE 6:**
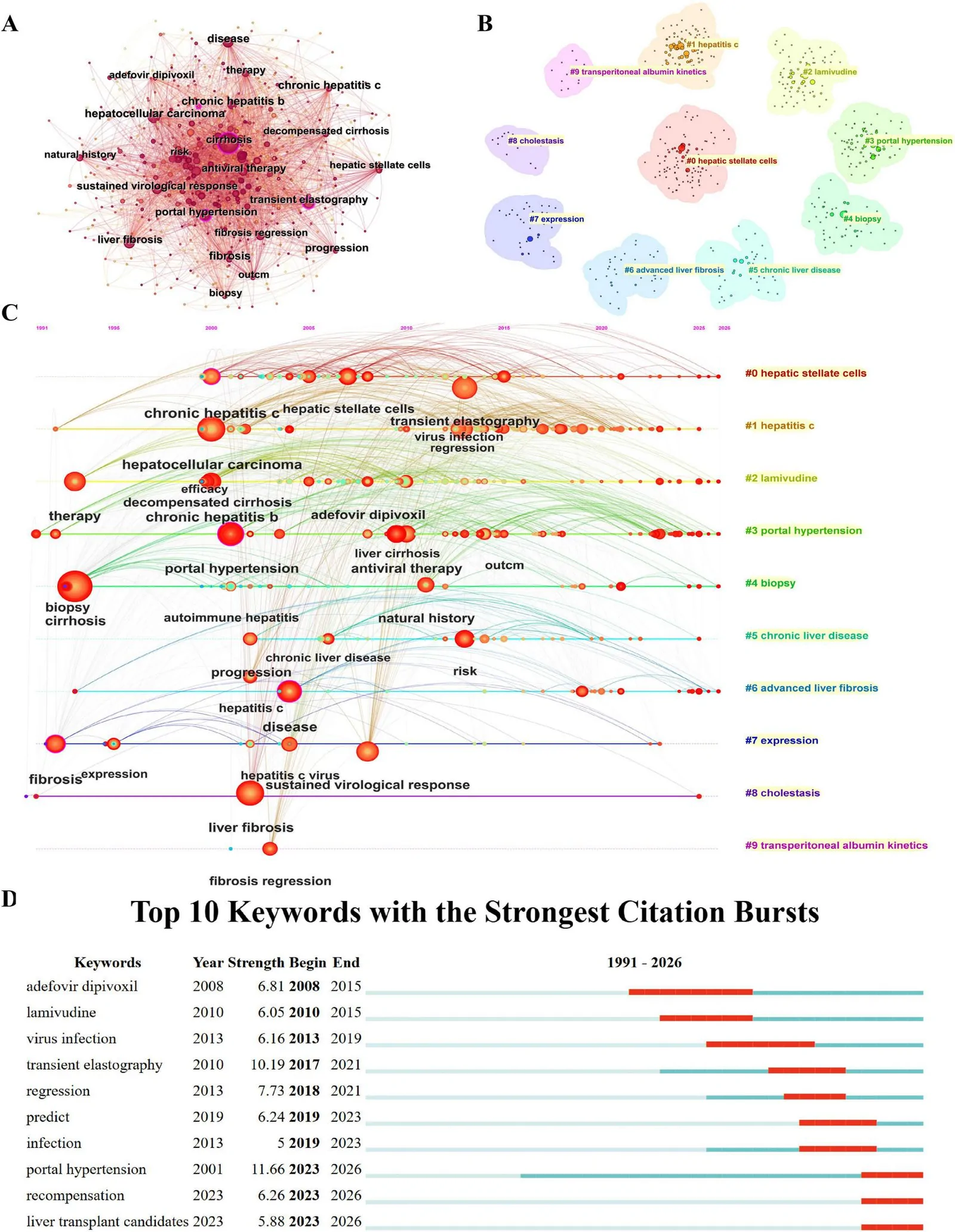
Keyword co-occurrence, clustering, timeline, and burst analysis in cirrhosis disease-modification and recompensation-related research. **(A)** Node size represents keyword frequency, links indicate co-occurrence within the same publication, and link width reflects co-occurrence strength. Time-coded colors represent the year of occurrence, and purple outer rings indicate betweenness centrality of at least 0.10. **(B)** Colors identify keyword clusters labelled using the log-likelihood ratio algorithm; cluster #0 is the largest cluster. **(C)** Keywords are positioned by year within their corresponding clusters, with node size representing frequency. **(D)** Red segments indicate periods of keyword citation bursts. Keyword frequencies used full counting, and a publication could contribute multiple keywords. Analyses were based on 588 WoSCC publications using the g-index with *k* = 25 and the stated pruning procedures.

The keyword co-occurrence network contained 660 keyword nodes, reflecting the thematic evolution of cirrhosis recompensation-related literature (Figure 6A). The most frequent keywords were cirrhosis (*n* = 140; centrality = 0.11), hepatocellular carcinoma (*n* = 108; centrality = 0.07), liver fibrosis (*n* = 107; centrality = 0.09), transient elastography (*n* = 77; centrality = 0.12), sustained virological response (*n* = 72; centrality = 0.03), chronic hepatitis B (*n* = 66; centrality = 0.08), fibrosis (*n* = 66; centrality = 0.10), portal hypertension (*n* = 63; centrality = 0.12), disease (*n* = 59; centrality = 0.10), and therapy (*n* = 55; centrality = 0.08). These terms indicate that the field was organized around viral hepatitis treatment, fibrosis regression, portal hypertension, and clinical outcome assessment (Supplementary Table 6).

Keyword clustering identified ten major clusters, including hepatic stellate cells, hepatitis C, lamivudine, portal hypertension, biopsy, chronic liver disease, advanced liver fibrosis, expression, cholestasis, and transperitoneal albumin kinetics (Figure 6B). These clusters corresponded broadly to antiviral therapy and virological response, fibrosis assessment and regression, portal hypertension and decompensation-related outcomes, and mechanistic studies of liver fibrosis. The timeline visualization further aligned with the three-stage trajectory (Figure 6C). In the first stage, keywords were mainly related to biopsy-based cirrhosis assessment, chronic viral hepatitis, natural history, and liver fibrosis. In the second stage after Chang TT 2010, antiviral agents, sustained virological response, transient elastography, fibrosis regression, and prediction-related terms became more prominent, reflecting the expansion of etiology-driven cirrhosis regression research. In the third stage after Baveno VII, portal hypertension, recompensation, and liver transplant candidates emerged as recent active topics.

Keyword burst analysis also supported this staged transition (Figure 6D). Early bursts were dominated by antiviral-related terms, including adefovir dipivoxil (2008–2015), lamivudine (2010–2015), and virus infection (2013–2019). Subsequent bursts included transient elastography (2017–2021), regression (2018–2021), predict (2019–2023), and infection (2019–2023). The current burst terms were portal hypertension (2023–2026; strength = 11.66), recompensation (2023–2026; strength = 6.26), and liver transplant candidates (2023–2026; strength = 5.88), indicating a recent shift from antiviral-driven fibrosis regression toward recompensation-oriented endpoints and advanced cirrhosis management (Supplementary Table 7).

### 3.7 Clinical evidence mapping within the WoSCC dataset and complementary PubMed analysis

**FIGURE 7:**
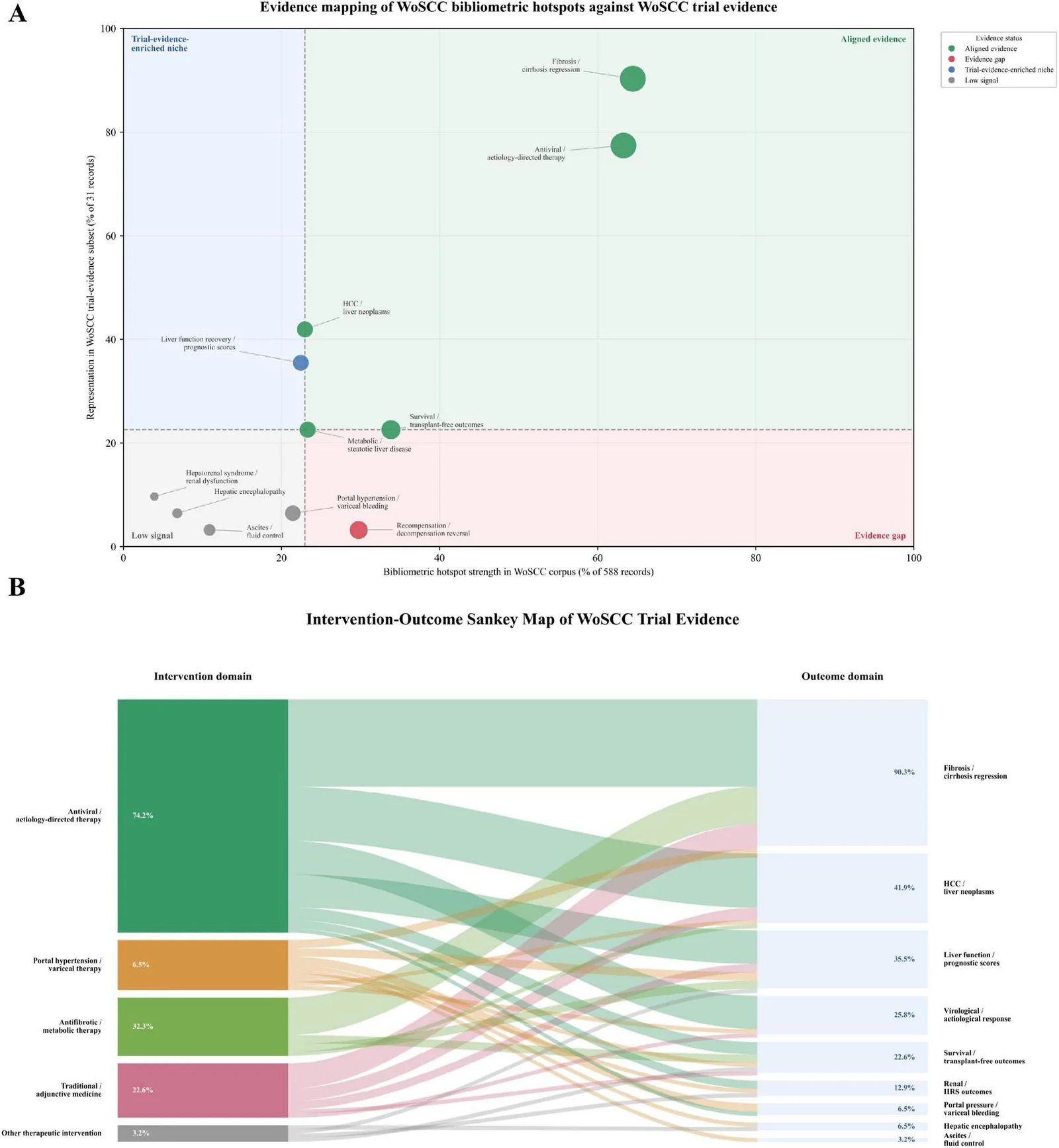
Clinical evidence mapping within the WoSCC dataset. **(A)** The evidence-gap matrix compares the prevalence of each clinical theme in the full WoSCC dataset (*x*-axis; *n* = 588) with its representation in the internally derived RCT subset (*y*-axis; *n* = 31). Dashed lines indicate the median proportions across the 11 mapped themes (22.96% and 22.58%, respectively). Bubble area was proportional to the number of WoSCC publications assigned to each theme marker area = (45 + 1.6n) points^2^, where n denotes the number of matched WoSCC publications), and colors indicate evidence status. **(B)** The Sankey map links intervention and outcome themes within the 31-publication RCT subset. Node percentages use 31 publications as the denominator, and edge widths are proportional to the number of publications assigned to each intervention–outcome pair. Themes were assigned non-exclusively; therefore, one publication could contribute to multiple themes and Sankey edges.

To examine whether bibliometric hotspots were represented in interventional evidence, we identified a 31-publication RCT subset within the 588-publication WoSCC dataset and applied the same non-exclusive clinical theme dictionary to both datasets. In the evidence gap matrix, fibrosis or cirrhosis regression and antiviral or etiology-directed therapy showed high representation in both the full WoSCC dataset and its RCT subset, placing them in the aligned-evidence region. HCC or liver neoplasms also showed relatively consistent representation, whereas liver function recovery or prognostic scores were more prominent in the RCT subset despite lower bibliometric hotspot strength. Survival or transplant-free outcomes and metabolic or steatotic liver disease were located near the evidence-status thresholds. By contrast, portal hypertension or variceal bleeding, hepatorenal syndrome or renal dysfunction, hepatic encephalopathy, and ascites or fluid control showed limited representation in both datasets. Recompensation or decompensation reversal had appreciable bibliometric hotspot strength but minimal representation in the RCT subset, constituting the clearest evidence gap (Figure 7A).

The intervention–outcome Sankey map further characterized the clinical focus of the WoSCC RCT subset. Because themes were assigned non-exclusively, individual publications could contribute to more than one intervention or outcome domain. Antiviral or etiology-directed therapy was the most frequently represented intervention domain (74.2%), followed by antifibrotic or metabolic therapy (32.3%) and traditional or adjunctive medicine (22.6%). The leading outcome domains were fibrosis or cirrhosis regression (90.3%), HCC or liver neoplasms (41.9%), liver function or prognostic scores (35.5%), virological or etiological response (25.8%), and survival or transplant-free outcomes (22.6%). No publication in the subset evaluated formal Baveno VII recompensation as an explicit trial endpoint. Thus, the within-WoSCC analysis showed that current interventional evidence remains concentrated on etiological control, fibrosis regression, liver-related outcomes, and conventional clinical endpoints, despite the growing bibliometric prominence of recompensation (Figure 7B). Complementary descriptive analysis of the 168 PubMed RCT publications showed a similar emphasis on antiviral therapy, fibrosis regression, liver function outcomes, and complication-directed management, while no publication evaluated formal Baveno VII recompensation as an explicit endpoint (Supplementary Figures 2A–D; Supplementary Table 9). This absence persisted among publications with follow-up beyond 48 weeks, suggesting that the endpoint gap could not be attributed solely to short follow-up (Supplementary Figures 4A–D).

## 4 Discussion

### 4.1 Principal findings: cirrhosis recompensation as an emerging disease-modification paradigm

This study provides a bibliometric and clinical evidence mapping overview of cirrhosis recompensation-related research. The main finding is that the field has evolved from a complication-control model toward a disease-modification model. This transition was not abrupt, but developed through three stages: an early period dominated by natural history, liver dysfunction, and complication management; a second period driven by etiology-directed therapy and fibrosis/cirrhosis regression; and a recent period centered on the formal concept of recompensation after the Baveno VII consensus.

Two landmark citation events structured this evolution. The 2010 entecavir study by Chang et al. provided histological evidence that advanced fibrosis and cirrhosis could regress during long-term antiviral therapy, challenging the traditional view that cirrhosis was largely irreversible (7). This study marked the transition from passive complication management to active disease modification. In contrast, the Baveno VII consensus in 2022 represented a conceptual turning point by defining recompensation as a clinically meaningful endpoint requiring etiological control, sustained absence of major decompensating events, and improvement in liver function. Our co-citation, burst, and keyword analyses consistently identified these two references as central temporal anchors (11). Importantly, these transition points were not supported by citation bursts alone. Stage-specific publication-rate comparisons and cumulative-trajectory analyses demonstrated significant and progressively larger increases in research output across the prespecified 2010 and 2022 boundaries, providing quantitative support for the three-stage framework.

A second important finding is that the bibliometric emergence of recompensation has outpaced its incorporation into interventional trial design. Although recompensation and decompensation reversal became recent hotspots, RCT evidence remained concentrated around antiviral response, fibrosis regression, survival, liver function, portal hypertension, hepatic encephalopathy, hepatorenal syndrome, and albumin-based interventions. No RCT in our mapped dataset directly used recompensation as an explicit endpoint. Therefore, recompensation has become an influential concept in scholarly discourse, but its translation into standardized clinical trial endpoints remains incomplete.

### 4.2 From cirrhosis irreversibility to disease modification: interpretation of the three-stage evolution

The three-stage trajectory identified in this study reflects a broader change in how cirrhosis is conceptualized. In the first stage, cirrhosis was mainly studied as an end-stage consequence of chronic liver injury. Research attention was directed toward natural history, prognostic scores, liver function, hepatocellular carcinoma, ascites, variceal bleeding, and hepatic encephalopathy. This reflected the clinical reality that cirrhosis was historically managed by preventing complications, treating acute decompensation, and selecting candidates for liver transplantation. At that time, reversal of cirrhosis was not a dominant research objective, and the term recompensation had not yet entered formal clinical vocabulary.

The second stage began after the accumulation of evidence that effective etiological treatment could modify the course of cirrhosis. Chang et al.’s 2010 entecavir study was a key turning point because it provided direct histological evidence that long-term viral suppression could be associated with regression of advanced fibrosis or cirrhosis (7). Subsequent studies in chronic hepatitis B and hepatitis C further strengthened the concept that sustained virological response, antiviral therapy, and fibrosis regression were linked to better clinical outcomes (8–10). The keyword evolution in our study, including sustained virological response, transient elastography, regression, and fibrosis, is consistent with this shift. Importantly, non-invasive fibrosis assessment expanded the field beyond paired biopsy studies and made longitudinal monitoring more feasible (22).

The third stage was triggered by the Baveno VII consensus, which gave the field a more clinically integrated endpoint (11). Recompensation is broader than fibrosis regression alone. It requires not only suppression or removal of the etiological driver, but also sustained clinical stability and improvement in liver function. This definition moved the field from asking whether liver scar tissue can regress to asking whether a decompensated patient can return to a more stable clinical state. Therefore, the recent surge in recompensation-related publications represents a maturation of the disease-modification paradigm rather than a simple increase in terminology (23–25).

### 4.3 Knowledge base and thematic transition: antiviral regression, portal hypertension, and recompensation

The co-cited reference and keyword analyses showed that the knowledge base of this field was built on several interconnected domains. The earliest influential domain was antiviral therapy, especially in chronic hepatitis B and hepatitis C (8, 9). References related to entecavir, tenofovir, sustained virological response, and histological regression formed the backbone of the second stage. These studies provided proof-of-concept evidence that removing or suppressing the causal injury could lead to structural and functional improvement in cirrhosis (26, 27).

A second domain involved fibrosis assessment and disease monitoring. Keywords such as liver fibrosis, transient elastography, biopsy, fibrosis regression, and prediction indicate that the field progressively shifted from static histological classification toward dynamic evaluation of disease evolution. This transition is important because recompensation cannot be captured by a single baseline measurement. It requires longitudinal assessment of etiology, portal hypertensive complications, liver function, and survival-related outcomes. Therefore, non-invasive tools and repeated clinical evaluation became essential for translating the idea of regression into patient-level disease modification (28, 29).

A third domain was portal hypertension. The recent burst of “portal hypertension” from 2023 to 2026 is particularly relevant. Recompensation is often discussed in relation to liver function recovery, but portal hypertension remains a central driver of decompensation events, including ascites, variceal bleeding, and hepatorenal syndrome (30, 31). The emergence of portal hypertension as a current hotspot suggests that the field is moving toward a more hemodynamic interpretation of recompensation. In this view, etiological control and fibrosis regression are necessary but may not be sufficient; sustained reduction in portal pressure and prevention of recurrent decompensation may also be required (32). This has direct implications for future studies evaluating non-selective beta-blockers, transjugular intrahepatic portosystemic shunt (TIPS), antiviral therapy, metabolic therapy, and antifibrotic interventions within a unified recompensation-oriented endpoint structure (33).

### 4.4 Global research landscape and interdisciplinary knowledge flow

The geographical and institutional results suggest that cirrhosis recompensation-related research has become a global field with both Western and Asian leadership. The United States and several European countries contributed substantially to early high-impact publications, consensus documents, and conceptual development. This is consistent with the prominent role of European hepatology networks in portal hypertension consensus building and with the high citation impact of journals such as Journal of Hepatology and Hepatology. These journals served not only as major publication venues but also as central sources of co-cited knowledge.

China has emerged as a major contributor in recent years. The rapid increase in Chinese publications, together with active institutional clusters such as Capital Medical University and the Chinese People’s Liberation Army General Hospital, indicates growing clinical and research capacity in cirrhosis regression and recompensation-related studies. This contribution may be partly related to the large burden of chronic hepatitis B and cirrhosis in China, which provides a strong clinical basis for studying antiviral therapy, fibrosis regression, and long-term outcomes (34, 35). The thematic comparison supported this interpretation: China-linked publications placed greater emphasis on HBV-related research and recompensation, whereas United States-linked publications more frequently addressed HCV and metabolic or steatotic liver disease. Fibrosis regression remained a shared priority, suggesting complementary opportunities for future collaboration.

The journal dual-map overlay further showed that this field is not confined to conventional clinical hepatology. Clinical medicine and hepatology journals served as the main citing domains, whereas the cited knowledge base extended to molecular biology, genetics, immunology, and broader biomedical sciences. This pattern reflects the translational nature of recompensation research. Clinically, recompensation is defined by patient-level outcomes, but biologically it is linked to fibrosis remodeling, inflammation resolution, extracellular matrix turnover, vascular remodeling, hepatocyte regeneration, metabolic injury, and portal hemodynamics (36–39). Therefore, future progress will likely depend on closer integration between clinical hepatology cohorts, mechanistic liver biology, imaging science, and trial methodology.

### 4.5 From bibliometric hotspots to clinical evidence: the unresolved RCT evidence gap

A distinctive feature of this study is the within-WoSCC comparison between bibliometric hotspots and the clinical endpoints evaluated in its RCT subset, complemented by a separate descriptive analysis of PubMed RCT evidence. This comparison does not establish therapeutic efficacy, but it provides clinical context for interpreting publication trends. The evidence gap matrix showed that antiviral or etiology-directed therapy, survival or transplant-free outcomes, and fibrosis/cirrhosis regression were positioned in the aligned-evidence quadrant. These themes were prominent both in the bibliometric corpus and in the RCT dataset, indicating that the second-stage disease-modification literature has a relatively mature clinical trial foundation.

In contrast, recompensation or decompensation reversal showed high bibliometric hotspot strength but minimal representation in the RCT dataset. This was the most evident evidence gap in our analysis. The Sankey map further illustrated how this gap was manifested. Current RCTs were mainly organized around antiviral therapy, hepatic encephalopathy therapy, hepatorenal syndrome therapy, albumin or volume expansion, portal hypertension or variceal therapy, antifibrotic or metabolic therapy, and other supportive interventions (40–42). Their outcomes were distributed across virological response, fibrosis regression, survival, liver function scores, renal outcomes, hepatic encephalopathy, portal pressure, and variceal bleeding. These outcomes are relevant to recompensation, but they do not equal formal recompensation as defined by Baveno VII.

This mismatch has important implications. It suggests that the third-stage concept of recompensation is currently more developed as a consensus-based and bibliometric hotspot than as a trial endpoint. Existing RCTs have tested components of the recompensation pathway, but rarely the integrated endpoint itself. This is understandable because Baveno VII was published recently, and designing trials around recompensation requires long follow-up, standardized event definitions, and careful patient selection. However, the complementary follow-up analysis showed that extended observation periods were common in the PubMed RCT dataset, while formal recompensation remained absent among publications with follow-up beyond 48 weeks. Therefore, this gap cannot be attributed to short follow-up alone and likely also reflects a temporal lag after Baveno VII and the continued use of individual clinical outcomes rather than a standardized recompensation endpoint. Nevertheless, the absence of direct recompensation endpoints means that current evidence cannot yet answer which interventions truly induce sustained recompensation rather than temporary improvement in isolated clinical parameters.

### 4.6 Future directions: toward recompensation-based trial design and standardized endpoints

Future research should move from descriptive recognition of recompensation toward prospective testing of recompensation-oriented strategies. First, endpoint standardization is essential. Trials should consider Baveno VII recompensation as a prespecified endpoint or key secondary endpoint, rather than relying only on isolated measures such as albumin, bilirubin, ascites control, hepatic encephalopathy recurrence, variceal bleeding, or Model for End-stage Liver Disease score. Because recompensation is a composite clinical state, endpoint adjudication should include etiology control, sustained absence of decompensating events, and objective liver function improvement over an adequate follow-up period.

Second, interventions should be interpreted through a mechanism-informed disease-modification lens. Antiviral therapy remains the clearest example of etiology-directed modification. However, future recompensation studies should also evaluate portal pressure reduction, metabolic therapy for steatotic liver disease, alcohol abstinence interventions, antifibrotic agents, gut–liver axis modulation, anti-inflammatory strategies, and TIPS in selected patients (39, 43, 44). These interventions may act at different points in the recompensation pathway: removing the causal injury, reducing portal pressure, promoting fibrosis remodeling, improving hepatocellular function, or preventing recurrent decompensation.

Third, longitudinal assessment should be strengthened. A transient reduction in ascites, improved laboratory score, or short-term freedom from bleeding should not be equated with recompensation. Future studies need durable follow-up, ideally beyond 12 months, to distinguish temporary stabilization from sustained clinical reversal. Repeated assessment of portal pressure, elastography, imaging-based vascular remodeling, biomarkers of extracellular matrix turnover, and patient-centered outcomes may help identify early predictors of true recompensation (36, 45).

Fourth, trial populations should be refined. Recompensation is unlikely to occur uniformly across all decompensated cirrhosis patients. Baseline etiology, degree of portal hypertension, liver reserve, inflammatory activity, sarcopenia, renal dysfunction, and prior decompensation burden may all modify the probability of response. Enriching trials for patients with modifiable etiology, preserved regenerative capacity, and measurable portal hypertensive risk may improve the ability to detect disease-modifying effects.

### 4.7 Strengths and limitations

Several limitations should be noted. First, the WoSCC-based corpus may have missed relevant studies indexed only in other databases, regional sources, or non-English literature. Accordingly, the English-language restriction may have introduced language-related selection bias and may limit the generalizability of the bibliometric and complementary RCT findings. Second, bibliometric indicators reflect publication activity and citation visibility, but do not directly measure study quality, biological validity, or clinical efficacy. Third, keyword clustering, burst detection, and rule-based theme matching are influenced by database indexing, author terminology, software parameters, and predefined dictionaries; therefore, the evidence gap matrix and Sankey map should be interpreted as exploratory evidence mapping rather than formal systematic review or meta-analysis. Finally, the PubMed RCT dataset was analysed separately from the WoSCC corpus and should be viewed as complementary clinical contextualization rather than a comprehensive assessment of all therapeutic evidence.

## Data Availability

The original contributions presented in this study are included in this article/Supplementary material, further inquiries can be directed to the corresponding authors.
