# Supplementary material for: From fibrosis regression to recompensation: bibliometric and clinical evidence mapping of disease modification in cirrhosis
Source: Front Med (Lausanne). 2026 Aug 20;13:1921542. doi: 10.3389/fmed.2026.1921542 (PMC13539136; doi:10.3389/fmed.2026.1921542)
Supplement: Supplementary file 1 [file Data_Sheet_1.pdf]

Supplementary Material

Article title: From fibrosis regression to recompensation: bibliometric and clinical evidence mapping of disease modification in cirrhosis

1 Supplementary Tables

Supplementary Table 1. Search strategy and eligibility criteria.

The search strategy was designed to capture disease-modification-related research in cirrhosis, because the term "cirrhosis recompensation" was formally defined only recently. All included PubMed publications were manually confirmed as primary reports of randomized controlled trials. Non-randomized controlled clinical trials were excluded.

A. Web of Science Core Collection search strategy

Database: Web of Science Core Collection. Search field: Topic (TS). Retrieval period: all available years up to May 31, 2026. Document and language limits were applied after retrieval.

Step	Theme	Search string	Use
#1	Direct recompensation terms	TS=("recompensation" OR "re-compensation" OR "recompensated" OR "re-compensated" OR "recompensate" OR "re-compensate")	Included in final WoSCC corpus
#2	Decompensation reversal terms	TS=("reversal of decompensation" OR "reverse decompensation" OR "reversed decompensation" OR "reversing decompensation" OR "reversibility of decompensation" OR "decompensation reversal" OR "decompensation reversed") AND TS=("cirrhosis" OR "liver" OR "hepatic" OR "hepato*")	Included in final WoSCC corpus
#3	Fibrosis/cirrhosis regression, reversal, resolution, or remodeling	TS=("fibrosis regression" OR "fibrosis reversal" OR "fibrosis resolution" OR "regression of fibrosis" OR "reversal of fibrosis" OR "resolution of fibrosis" OR "cirrhosis regression" OR "cirrhosis reversal" OR "cirrhosis resolution" OR "regression of cirrhosis" OR "reversal of cirrhosis" OR "resolution of cirrhosis" OR "hepatic remodeling" OR "hepatic remodelling" OR "liver remodeling" OR "liver remodelling") AND TS=("cirrhosis" OR "decompensat*" OR "advanced liver disease" OR "chronic liver disease" OR "portal hypertension")	Included in final WoSCC corpus
#4	Symptom resolution, transplant delisting or broad clinical improvement	TS=((ascites NEAR/2 (resolution OR disappear* OR resolv* OR free OR clearance OR eliminat*)) OR (encephalopathy NEAR/2 (resolution OR reversal OR resolv* OR revers* OR remission)) OR (variceal NEAR/3 (resolution OR rebleed* OR prevention OR eliminat*))) AND TS=(cirrhosis OR decompensat* OR "liver disease" OR hepatic) AND TS=(antiviral OR DAA* OR "direct-acting" OR entecavir OR tenofovir OR SVR OR "sustained virolog*" OR abstinence OR etiolog* OR aetiolog* OR treatment OR therapy) OR TS=("delisting" OR "delisted" OR "delist" OR "waitlist removal" OR "waiting list removal" OR "waitlist improvement" OR "transplant-free survival" OR "transplant free survival" OR "clinical improvement" OR "clinical recovery") AND TS=("cirrhosis" OR "decompensated liver" OR "liver transplant*" OR "hepatic decompensation")	Included in final WoSCC corpus
#5	Liver function recovery or improvement	TS=("liver function improvement" OR "liver function restoration" OR "liver function recovery" OR "hepatic function improvement" OR "hepatic function restoration" OR "hepatic function recovery" OR "MELD improvement" OR "MELD score improvement" OR "MELD decrease" OR "Child-Pugh improvement" OR "CTP improvement" OR "CTP score	Included in final WoSCC corpus

Step	Theme	Search string	Use
		improvement") AND TS=("decompensated cirrhosis" OR "decompensated liver" OR "hepatic decompensation" OR "advanced cirrhosis")	
#6	Final WoSCC combination	#1 OR #2 OR #3 OR #4 OR #5	Final bibliometric retrieval set before screening

B. PubMed search strategy for RCT evidence mapping

Database: PubMed. Retrieval period: up to May 31, 2026. The final PubMed RCT dataset was derived from a broader clinical evidence search and then screened for relevance to cirrhosis disease modification, regression/reversal, liver-function recovery, portal-hypertension control, and decompensation-related outcomes. The PubMed dataset was analysed independently from the WoSCC bibliometric corpus.

Search	Search string	Purpose
Broader clinical evidence search used for final RCT screening	("cirrhosis" OR "decompensated cirrhosis" OR "portal hypertension") AND ("recompensation" OR "reversal" OR "regression" OR "recovery" OR "resolution" OR "improvement"[Title/Abstract] OR "remission"[Title/Abstract]) AND ("randomized controlled trial" OR "RCT" OR "randomized" OR "controlled trial") AND ("treatment" OR "therapy" OR "intervention")	Used to derive the final PubMed RCT dataset after screening for cirrhosis disease modification, regression/reversal, liver-function recovery, portal-hypertension control, and decompensation-related outcomes

C. Eligibility criteria for the WoSCC bibliometric corpus

Category	Criteria
Inclusion criteria	(1) Records retrieved from the WoSCC using the final topic-search strategy; (2) publications indexed from database inception to May 31, 2026; (3) original articles or reviews; (4) English-language publications; (5) studies related to cirrhosis, decompensated cirrhosis, advanced chronic liver disease, portal hypertension, fibrosis/cirrhosis regression or resolution, hepatic remodeling/remodelling, recompensation, decompensation reversal, symptom resolution under therapeutic or aetiological control, or liver-function recovery; (6) records belonging to Web of Science subject categories relevant to gastroenterology, hepatology, liver disease, or related biomedical research after category screening; and (7) records with sufficient bibliographic and citation information for bibliometric analysis.
Exclusion criteria	(1) Duplicate records; (2) document types other than articles or reviews, such as meeting abstracts, editorials, letters, corrections, news items, book chapters, or proceedings papers; (3) non-English publications; (4) records outside relevant Web of Science subject categories after screening; (5) studies not focused on cirrhosis, advanced chronic liver disease, portal hypertension, fibrosis/cirrhosis regression, recompensation, or disease modification; (6) records addressing general clinical improvement, transplant delisting, or survival outcomes without a clear relationship to cirrhosis regression, recompensation, aetiological control, or liver-function recovery; (7) studies focused only on non-hepatic diseases, non-cirrhotic fibrosis, acute liver injury without chronic cirrhosis context, or unrelated organ systems; and (8) records judged off-topic after title and abstract screening.

D. Eligibility criteria for the PubMed RCT evidence-mapping dataset

Category	Criteria
Inclusion criteria	(1) Records retrieved through the broader PubMed clinical evidence search used for final RCT screening; (2) publications up to May 31, 2026; (3) English-language publications; (4) randomized controlled trials involving

Category	Criteria
	patients with cirrhosis, decompensated cirrhosis, portal hypertension, or cirrhosis-related chronic liver disease; (5) evaluation of a therapeutic or disease-modifying intervention, including antiviral therapy, aetiology-directed treatment, pharmacological therapy, portal hypertension-directed therapy, complication-directed treatment, lifestyle or abstinence-related intervention, or other clinical interventions relevant to cirrhosis outcomes; (6) reporting of outcomes conceptually related to recompensation, cirrhosis regression or reversal, fibrosis regression, ascites resolution, variceal bleeding control, hepatic encephalopathy improvement, portal hypertension improvement, liver-function recovery, MELD or Child-Pugh improvement, transplant-free survival, or other cirrhosis-related clinical improvement.
Exclusion criteria	(1) Non-randomized studies, observational studies, case reports, case series, reviews, meta-analyses, editorials, letters, protocols without results, animal studies, or in vitro studies; (2) non-English publications; (3) studies not involving cirrhosis, decompensated cirrhosis, portal hypertension, or a clearly relevant chronic liver disease population; (4) mixed-population studies in which cirrhosis-related data could not be distinguished; (5) studies without a therapeutic or interventional component, such as purely diagnostic, prognostic, biomarker, epidemiological, or mechanistic studies; (6) studies lacking outcomes relevant to recompensation, regression, reversal, portal hypertension, decompensation events, or liver-function recovery.

E. Screening and data cleaning procedure

Records retrieved from the WoSCC and PubMed were exported and processed separately in EndNote 21. Duplicates were checked using digital object identifiers, titles, publication years, and author information, followed by manual verification when necessary. Two reviewers independently screened titles and abstracts according to predefined criteria, and disagreements were resolved through discussion with a third reviewer. Full texts were reviewed when eligibility could not be determined from titles and abstracts. The final WoSCC and PubMed datasets were then cleaned and harmonized before bibliometric analysis and evidence mapping.

Supplementary Table 2. Publication dynamics across bibliometric stages

Transition point	Comparison	Before publication	After publication	Before rate	After rate	Rate ratio (95% CI)	P value
2010	<=2010 vs 2011-2022	114	281	1.9/year	23.4/year	12.12 (9.75 to 15.07)	**<0.001**
2022	2011-2022 vs 2023-2025	281	150	23.4/year	50.0/year	2.14 (1.75 to 2.60)	**<0.001**

Note. Publication rates were calculated as records per year. The recent-stage rate was calculated for 2023-2025 because 2026 was incomplete at the time of retrieval. Rate ratios and 95% confidence intervals were estimated using Poisson rate tests.

Supplementary Table 3. Top 10 countries/regions by publication output and collaboration indicators in the WoSCC corpus.

Country/region	Articles	Articles (%)	SCP	MCP	MCP (%)
United States	141	24.0	67	74	52.5
China	135	23.0	103	32	23.7
Germany	71	12.1	41	30	42.3
Spain	61	10.4	25	36	59.0

Country/region	Articles	Articles (%)	SCP	MCP	MCP (%)
United Kingdom	51	8.7	23	28	54.9
Italy	50	8.5	28	22	44
France	46	7.8	23	23	50
Canada	26	4.4	4	22	84.6
Australia	21	3.6	3	18	85.7
India	21	3.6	11	10	47.6

Note. Percentages are based on the 588-record WoSCC corpus. SCP, single-country publication; MCP, multiple-country publication. Country/region counts are not additive because full counting was used; one article may contribute to more than one country/region.

Supplementary Table 4. Comparison of research themes between China and the United States.

Research theme	China publications, n/N (%)	United States publications, n/N (%)	Difference, percentage points	P value	Relative emphasis
HBV-related research	83/135 (61.5)	47/141 (33.3)	+28.1	**<0.001**	Higher in China
HCV-related research	11/135 (8.1)	33/141 (23.4)	-15.3	**0.002**	Higher in United States
Alcohol-related liver disease	3/135 (2.2)	10/141 (7.1)	-4.9	0.083	No clear difference
Metabolic or steatotic liver disease	14/135 (10.4)	37/141 (26.2)	-15.9	**<0.001**	Higher in United States
Fibrosis or cirrhosis regression	87/135 (64.4)	103/141 (73.0)	-8.6	0.139	No clear difference
Portal hypertension or variceal management	18/135 (13.3)	27/141 (19.1)	-5.8	0.250	No clear difference
Decompensation and complication-related outcomes	37/135 (27.4)	25/141 (17.7)	+9.7	0.083	No clear difference
Liver function or prognostic assessment	37/135 (27.4)	26/141 (18.4)	+9.0	0.083	No clear difference
HCC or liver neoplasms	31/135 (23.0)	33/141 (23.4)	-0.4	1.000	No clear difference
Recompensation or decompensation reversal	42/135 (31.1)	24/141 (17.0)	+14.1	**0.007**	Higher in China
Traditional Chinese medicine	11/135 (8.1)	3/141 (2.1)	+6.0	**0.007**	Higher in China

*Note. Themes were assigned non-exclusively by rule-based matching of titles, abstracts, author keywords, and Keywords Plus. Country-linked profiles used full counting (China, n=135; United States, n=141); 24 bilateral publications were counted in both profiles. Difference was calculated as China minus the United States. *To address the non-independence introduced by bilateral publications, two-sided Fisher exact tests were performed after excluding these publications (China only, n=111; United States only, n=117) and adjusted across the 11 themes using the Benjamini-Hochberg procedure. Positive values indicate higher representation among China-linked publications. HBV, hepatitis B virus; HCV, hepatitis C virus; HCC, hepatocellular carcinoma.*

Supplementary Table 5. Top co-cited references in cirrhosis disease-modification and recompensation-related research.

Reference	Source	Co-citations	DOI
de Franchis R, 2022	J HEPATOL	84	10.1016/j.jhep.2021.12.022
Wang Q, 2022	J HEPATOL	45	10.1016/j.jhep.2022.07.037
Marcellin P, 2013	LANCET	38	10.1016/S0140-6736(12)61425-1
Hofer BS, 2023	LIVER INT	36	10.1111/liv.15676
Bachofner JA, 2017	LIVER INT	28	10.1111/liv.13256
Reiberger T, 2023	DIGEST LIVER DIS	24	10.1016/j.dld.2022.12.014
Chang TT, 2010	HEPATOLOGY	24	10.1002/hep.23785
Kim TH, 2022	ALIMENT PHARM THER	23	10.1111/apt.16658
Pose E, 2021	J HEPATOL	23	10.1016/j.jhep.2021.02.033
Hui VWK, 2023	JHEP REP	22	10.1016/j.jhepr.2023.100814
DAmbrosio R, 2012	HEPATOLOGY	19	10.1002/hep.25606
Lens S, 2020	J HEPATOL	19	10.1016/j.jhep.2020.05.050
Knop V, 2016	J VIRAL HEPATITIS	18	10.1111/jvh.12578
Ginès P, 2021	LANCET	18	10.1016/S0140-6736(21)01374-X
Premkumar M, 2024	GASTROENTEROLOGY	17	10.1053/j.gastro.2024.08.018
Kisseleva T, 2021	NAT REV GASTRO HEPAT	17	10.1038/s41575-020-00372-7
Harrison SA, 2024	NEW ENGL J MED	15	10.1056/NEJMoa2309000
EASL–ALEH, 2015	J HEPATOL	15	10.1016/j.jhep.2015.04.006
Sanyal AJ, 2022	HEPATOLOGY	15	10.1002/hep.32204
Mandorfer M, 2020	HEPATOLOGY	14	10.1002/hep.30885

Note. Co-citation counts, source, year, and DOI were taken from the CiteSpace co-cited reference node output to match the co-citation network reported in the main text.

Supplementary Table 6. Top keywords by frequency and centrality in the WoSCC corpus.

Rank	Keyword	Frequency	Centrality	First year
1	Cirrhosis	140	0.11	1993
2	Hepatocellular Carcinoma	108	0.07	2000
3	Liver Fibrosis	107	0.09	2008
4	Transient Elastography	77	0.12	2010
5	Sustained Virological Response	72	0.03	2008
6	Chronic Hepatitis B	66	0.08	2000
7	Fibrosis	66	0.1	1992
8	Portal Hypertension	63	0.12	2001
9	Disease	59	0.1	2004
10	Therapy	55	0.08	1993

Rank	Keyword	Frequency	Centrality	First year
11	Regression	52	0.02	2013
12	Chronic Hepatitis C	50	0.1	2000
13	Antiviral Therapy	50	0.06	2010
14	Risk	46	0.09	2013
15	Hepatic Stellate Cells	42	0.07	2007
16	Liver Cirrhosis	39	0.05	2010
17	Decompensated Cirrhosis	37	0.09	2000
18	Natural History	33	0.06	2011
19	Hepatitis C Virus	32	0.02	2004
20	Fibrosis Regression	31	0.03	2003
21	Progression	28	0.09	2006
22	Virus Infection	26	0.02	2013
23	Biopsy	26	0.03	1993
24	Infection	25	0.01	2013
25	Hepatitis C	24	0.02	2002
26	Nonalcoholic Steatohepatitis	23	0.03	2015
27	Management	21	0.02	2008
28	Efficacy	20	0.01	2000
29	Diagnosis	20	0.02	2010
30	Hepatitis B	19	0.02	2004

Note. Keyword frequency, centrality, and first year were taken from the CiteSpace keyword node output to match the keyword co-occurrence network reported in the main text.

Supplementary Table 7. Keyword clusters and burst terms in the WoSCC corpus.

A. Keyword clusters

Cluster ID	Size	Silhouette	Mean year	LSI label	LLR label
#0	97	0.709	2010	liver fibrosis; chronic hepatitis c; chronic hepatitis b; antiviral therapy; hepatic stellate cell	hepatic stellate cells; hepatic stellate cell; macrophage; extracellular matrix; recompensation
#1	88	0.648	2014	hepatocellular carcinoma; hepatitis c; sustained virologic response; direct-acting antiviral agents; daa therapy	hepatitis c; liver biopsy; sustained virological response; sustained virologic response; recompensation
#2	82	0.764	2010	chronic hepatitis b; liver fibrosis; clinical trial; ruangan granule; sustained virological response	lamivudine; hepatitis b virus; chronic hepatitis b; decompensated cirrhosis; adefovir dipivoxil

Cluster ID	Size	Silhouette	Mean year	LSI label	LLR label
#3	71	0.678	2017	portal hypertension; variceal bleeding; transjugular intrahepatic portosystemic shunt; liver cirrhosis; chronic liver disease	portal hypertension; hepatic encephalopathy; recompensation; acute-on-chronic liver failure; ascites
#4	49	0.826	2006	cirrhosis; biopsy; survival; liver disease; regression	biopsy; autoimmune hepatitis; portal hypertension; hepatitis; features
#5	41	0.822	2015	liver stiffness; transient elastography; direct-acting antivirals; sustained virologic response; additive mixed model	chronic liver disease; metabolic syndrome; fatty liver disease; cardiovascular disease; recompensation
#6	35	0.787	2018	liver fibrosis; diabetes mellitus; direct-acting antivirals; chronic hepatitis b; pharmacological therapies	advanced liver fibrosis; baveno vii; damps; invasion; alcohol-related cirrhosis
#7	34	0.915	2002	hepatocellular carcinoma; fibrosis; transient elastography; risk; infection	expression; ppar gamma; toll-like receptors; fibroblasts; metabolomics
#8	13	0.995	1993	liver failure; gastrointestinal bleeding; renal failure; prognostic nutritional index; liver cirrhosis	cholestasis; renal failure; encephalopathy; prognostic nutritional index; nutrition
#9	12	1.000	2001	albumin; ascites; cirrhosis; diuretic-resistant ascites; transperitoneal albumin kinetics	transperitoneal albumin kinetics; diuretic-resistant ascites; transvascular albumin transport; albumin; ascites

B. Burst terms

Keyword	Strength	Begin	End	Duration	Related stage
Recompensation	6.26	2023	2026	4	Recent stage (2023-2026)
Portal Hypertension	11.66	2023	2026	4	Recent stage (2023-2026)
Regression	7.72	2018	2021	4	Middle stage (2011-2022)
Transient Elastography	7.28	2017	2021	5	Middle stage (2011-2022)
Liver Transplant Candidates	5.88	2023	2026	4	Recent stage (2023-2026)
Natural History	6.25	2011	2014	4	Middle stage (2011-2022)
Liver Stiffness	5.82	2018	2018	1	Middle stage (2011-2022)
Adefovir Dipivoxil	5.79	2008	2015	8	Early stage (up to 2010); Middle stage (2011-2022)
Hepatic Stellate Cells	5.69	2015	2017	3	Middle stage (2011-2022)
Biopsy	5.65	2017	2019	3	Middle stage (2011-2022)
Lamivudine	5.17	2010	2015	6	Early stage (up to 2010); Middle stage (2011-2022)
Oral Antiviral Therapy	4.49	2024	2025	2	Recent stage (2023-2026)
Cirrhosis	4.47	2018	2019	2	Middle stage (2011-2022)
Simple Noninvasive Index	4.46	2021	2022	2	Middle stage (2011-2022)
Advanced Chronic Liver Disease	4.40	2023	2024	2	Recent stage (2023-2026)

Keyword	Strength	Begin	End	Duration	Related stage
Liver Fibrosis	4.22	2018	2019	2	Middle stage (2011-2022)
Tenofovir Disoproxil Fumarate	4.13	2014	2015	2	Middle stage (2011-2022)
Virus Infection	4.01	2013	2019	7	Middle stage (2011-2022)
Meta-analysis	3.93	2016	2016	1	Middle stage (2011-2022)
Transplant Candidates	3.69	2025	2026	2	Recent stage (2023-2026)
Decompensation	3.66	2023	2024	2	Recent stage (2023-2026)
Liver Cirrhosis	3.45	2026	2026	1	Recent stage (2023-2026)
Follow Up	3.39	2000	2000	1	Early stage (up to 2010)
Predict	3.38	2019	2023	5	Middle stage (2011-2022); Recent stage (2023-2026)
Fibrosis	3.36	2015	2016	2	Middle stage (2011-2022)
Decompensated Cirrhosis	3.36	2024	2025	2	Recent stage (2023-2026)
Accuracy	3.34	2014	2014	1	Middle stage (2011-2022)
Fatty Liver Disease	3.34	2021	2022	2	Middle stage (2011-2022)
Inflammation	3.33	2019	2020	2	Middle stage (2011-2022)
Sinusoidal Endothelial Cells	3.27	2015	2015	1	Middle stage (2011-2022)

Note. Keyword clustering and burst detection were performed on cleaned WoSCC keyword data. The related stages correspond to the temporal framework used in the main text: Early stage: up to 2010; Middle stage: 2011-2022; Recent stage: 2023-2026. Cluster labels were automatically generated by CiteSpace and should be interpreted descriptively, especially for clinically peripheral labels produced by algorithmic co-occurrence clustering.

Supplementary Table 8. Operational definitions and representative matching terms for clinical themes.

Domain	Theme	Operational definition	Representative matching terms
**A. Clinical themes used in the evidence-gap matrix**			
Evidence theme	Fibrosis / cirrhosis regression	Regression, reversal, or improvement of hepatic fibrosis or cirrhosis assessed histologically or by validated non-invasive measures.	fibrosis regression; cirrhosis regression; fibrosis reversal; cirrhosis reversal; liver fibrosis; hepatic fibrosis; histological/histologic improvement or regression; transient elastography; liver stiffness; Ishak; METAVIR; collagen; ELF test; FibroScan
	Antiviral / aetiology-directed therapy	Therapy targeting viral hepatitis or another underlying cause of liver disease, including documented virological control.	antiviral; entecavir; tenofovir; lamivudine; adefovir; telbivudine; interferon; peginterferon; nucleoside/nucleotide/nucleos(t)ide analogue; HBV; HCV; HDV; hepatitis B/C/D; sustained virological/virologic response; viral suppression; DAA; direct-acting antiviral; HBsAg; HBeAg
	Survival / transplant-free outcomes	Mortality, overall or transplant-free survival, transplantation, delisting, or major liver-related clinical events.	survival; mortality; death; transplant-free; liver transplantation; transplantation; delisting; prognosis; clinical outcomes; liver-related event; liver-related clinical event
	Recompensation / decompensation reversal	Explicit recompensation or sustained reversal/resolution of prior decompensation. Formal recompensation required record-level confirmation of an explicit Baveno VII-compatible endpoint.	recompensation; recompensated; re-compensation; decompensation reversal; stable improvement; resolution of decompensation; Baveno VII
	Metabolic / steatotic liver disease	Metabolic dysfunction, steatosis, or steatohepatitis as the disease context or therapeutic target.	NAFLD; NASH; MASLD; MASH; steatosis; steatohepatitis; steatotic liver disease; obesity; diabetes; metabolic dysfunction; metabolic dysfunction-associated

Domain	Theme	Operational definition	Representative matching terms
	HCC / liver neoplasms	Hepatocellular carcinoma or other primary hepatic neoplastic outcomes.	hepatocellular carcinoma; HCC; liver cancer; liver neoplasm; hepatic neoplasm
	Liver function recovery / prognostic scores	Improvement or assessment of hepatic function, biochemical recovery, or validated prognostic scores.	liver function; hepatic function; albumin; bilirubin; MELD; Child-Pugh; Child-Turcotte-Pugh; prognostic score; ALT normalization; alanine aminotransferase
	Portal hypertension / variceal bleeding	Portal-pressure reduction, portal hypertension control, or prevention/treatment of variceal bleeding.	portal hypertension; portal pressure; HVPG; variceal bleeding; varices; rebleeding; non-selective beta-blocker; NSBB; carvedilol; TIPS; transjugular intrahepatic
	Ascites / fluid control	Resolution or control of ascites and related fluid-management outcomes.	ascites; paracentesis; diuretic; fluid control; refractory ascites
	Hepatic encephalopathy	Overt or minimal hepatic encephalopathy and related cognitive outcomes.	hepatic encephalopathy; minimal hepatic encephalopathy; encephalopathy; cognitive outcome; psychometric
	Hepatorenal syndrome / renal dysfunction	Hepatorenal syndrome, acute kidney injury, or other clinically relevant renal dysfunction.	hepatorenal syndrome; HRS; renal dysfunction; renal failure; acute kidney injury; AKI; creatinine; terlipressin; vasoconstrictor
**B. Intervention domains used in the intervention-outcome Sankey map**			
Intervention	Antiviral / aetiology-directed therapy	Antiviral treatment or another intervention directed at the underlying liver-disease aetiology.	antiviral; entecavir; tenofovir; lamivudine; adefovir; telbivudine; interferon; peginterferon; nucleoside/nucleotide/nucleos(t)ide analogue; DAA; direct-acting antiviral; viral suppression
	Portal hypertension / variceal therapy	Pharmacological, endoscopic, or shunt-based treatment of portal hypertension or varices.	portal pressure; portal hypertension; HVPG; variceal; varices; TIPS; transjugular intrahepatic; beta-blocker; carvedilol; propranolol; nadolol; band ligation; sclerotherapy
	Albumin / volume expansion	Albumin administration or other plasma-volume expansion.	albumin administration; albumin infusion; volume expansion; plasma expander
	Hepatic encephalopathy therapy	Therapy intended to prevent or treat overt or minimal hepatic encephalopathy.	rifaximin; lactulose; probiotic; synbiotic; ammonia-lowering; minimal hepatic encephalopathy
	Vasoconstrictor / HRS therapy	Vasoconstrictor-based treatment of hepatorenal syndrome or cirrhosis-associated renal dysfunction.	terlipressin; vasoconstrictor; noradrenaline; norepinephrine; midodrine; octreotide; HRS-AKI
	Antifibrotic / metabolic therapy	Direct antifibrotic treatment or therapy targeting metabolic/steatotic liver disease.	antifibrotic; lanifibranor; selonsertib; cilofexor; firsocostat; hydronidone; simtuzumab; pan-PPAR; PPAR agonist; NASH; MASH; NAFLD; MASLD; metabolic; farglitazar; fazirsiran
	Traditional / adjunctive medicine	Traditional medicine, herbal therapy, or adjunctive treatment not included in the principal intervention domains.	traditional Chinese medicine; Biejia; Biejia-Ruangan; Ruangan; Fuzheng; Huayu; Fuzheng Huayu; salvianolic; An-Luo-Hua-Xian; Huangjia; herbal; Chinese herbal
	Other therapeutic intervention	Therapeutic intervention that did not match any prespecified intervention domain after record-level review.	Assigned only when no prespecified intervention theme was matched
**C. Outcome domains used in the intervention-outcome Sankey map and PubMed descriptive analysis**			
Outcome	Formal Baveno VII recompensation	An explicit trial endpoint consistent with formal Baveno VII recompensation rather than an isolated improvement in one complication.	recompensation; recompensated; Baveno VII recompensation; assignment required record-level manual confirmation
	Virological / aetiological response	Virological suppression, antigen loss, or another measurable response to aetiology-directed treatment.	viral load; HBV DNA; HCV RNA; HDV RNA; SVR; sustained virological/virologic response; HBsAg; HBeAg; viral suppression; virological/virologic response
	Fibrosis / cirrhosis regression	Histological or validated non-invasive evidence of improvement in fibrosis or cirrhosis.	fibrosis regression; cirrhosis regression; fibrosis reversal; cirrhosis reversal; fibrosis stage; liver stiffness; histological/histologic regression or improvement; Ishak; METAVIR; collagen; FibroScan; ELF test
	Portal pressure / variceal bleeding	Change in portal pressure or occurrence/prevention of variceal bleeding.	HVPG; portal pressure; portal hypertension; variceal bleeding; varices; rebleeding
	Hepatic encephalopathy	Occurrence, resolution, recurrence, or cognitive consequences of overt or minimal hepatic encephalopathy.	hepatic encephalopathy; minimal hepatic encephalopathy; encephalopathy; cognitive outcome; psychometric
	Renal / HRS outcomes	Renal-function outcomes, acute kidney injury, or hepatorenal syndrome response.	hepatorenal syndrome; HRS; creatinine; renal; acute kidney injury; AKI

Domain	Theme	Operational definition	Representative matching terms
	Liver function / prognostic scores	Biochemical liver-function measures or validated prognostic scores.	MELD; Child-Pugh; Child-Turcotte-Pugh; albumin; bilirubin; liver function; hepatic function; ALT normalization; alanine aminotransferase; CTP; prognosis/prognostic
	Survival / transplant-free outcomes	Mortality, survival, transplant-free survival, transplantation, or major liver-related events.	survival; mortality; death; transplant-free; liver transplantation; clinical outcomes; liver-related event; HCC-free survival
	HCC / liver neoplasms	Development, recurrence, or prevention of hepatocellular carcinoma or other hepatic neoplasms.	hepatocellular carcinoma; HCC; liver cancer; liver neoplasm; hepatic neoplasm
	Ascites / fluid control	Resolution, recurrence, or control of ascites and related fluid-management outcomes.	ascites; paracentesis; fluid control; diuretic
	Other clinical outcome	Clinically relevant outcome that did not match any prespecified outcome domain after record-level review.	Assigned only when no prespecified outcome theme was matched

Note. Matching was case-insensitive, and terms within each theme were combined using OR logic. WoSCC matching used titles (TI), abstracts (AB), author keywords (DE), and Keywords Plus (ID); PubMed matching used titles, abstracts, and the available keyword field (DE). A publication was assigned provisionally to every matching theme, and ambiguous assignments were checked at the record level. Themes were non-exclusive. "Other therapeutic intervention" or "Other clinical outcome" was assigned only when no prespecified theme was matched. Formal Baveno VII recompensation required explicit record-level confirmation and was not inferred from an isolated mention of decompensation or improvement in a single complication. DAA, direct-acting antiviral; ELF, enhanced liver fibrosis; HCC, hepatocellular carcinoma; HRS, hepatorenal syndrome; HVPG, hepatic venous pressure gradient; RCT, randomized controlled trial.

Supplementary Table 9. PubMed RCT publications and assigned intervention and outcome themes.

No.	PMID / DOI	Intervention theme(s)	Outcome theme(s)
1	PMID: 41317314<br>DOI: 10.1002/ueg2.70153	Antiviral / aetiology-directed therapy	Virological / aetiological response; Liver function / prognostic scores; Survival / transplant-free outcomes
2	PMID: 41200852<br>DOI: 10.1111/liv.70399	Portal hypertension / variceal therapy; Vasoconstrictor / HRS therapy	Portal pressure / variceal bleeding; Renal / HRS outcomes; Liver function / prognostic scores; Survival / transplant-free outcomes; Ascites / fluid control
3	PMID: 41138575<br>DOI: 10.1016/j.phymed.2025.157434	Antiviral / aetiology-directed therapy; Traditional / adjunctive medicine	Fibrosis / cirrhosis regression
4	PMID: 40891155<br>DOI: 10.1111/dom.70071	Antifibrotic / metabolic therapy	Other clinical outcome
5	PMID: 40757786<br>DOI: 10.1111/liv.70257	Other therapeutic intervention	Liver function / prognostic scores; Survival / transplant-free outcomes
6	PMID: 40658810<br>DOI: 10.1097/HC9.0000000000000766	Vasoconstrictor / HRS therapy	Renal / HRS outcomes
7	PMID: 40576034<br>DOI: 10.1161/JAHA.124.040848	Other therapeutic intervention	Liver function / prognostic scores; Survival / transplant-free outcomes
8	PMID: 40370077<br>DOI: 10.1111/dom.16443	Antifibrotic / metabolic therapy	Other clinical outcome
9	PMID: 40318534<br>DOI: 10.1016/j.phymed.2025.156741	Antiviral / aetiology-directed therapy; Traditional / adjunctive medicine	Fibrosis / cirrhosis regression; Liver function / prognostic scores
10	PMID: 40053863<br>DOI: 10.1097/HEP.0000000000001295	Vasoconstrictor / HRS therapy	Renal / HRS outcomes; Survival / transplant-free outcomes

No.	PMID / DOI	Intervention theme(s)	Outcome theme(s)
11	PMID: 39702655<br>DOI: 10.1007/s12072-024-10764-5	Antiviral / aetiology-directed therapy	Virological / aetiological response; Liver function / prognostic scores
12	PMID: 39683419<br>DOI: 10.3390/nu16234024	Hepatic encephalopathy therapy; Antifibrotic / metabolic therapy	Hepatic encephalopathy
13	PMID: 39672198<br>DOI: 10.1055/a-2484-0747	Other therapeutic intervention	Other clinical outcome
14	PMID: 39373164<br>DOI: 10.1111/jvh.14012	Antiviral / aetiology-directed therapy	Virological / aetiological response; Fibrosis / cirrhosis regression
15	PMID: 39248039<br>DOI: 10.1111/liv.16092	Antifibrotic / metabolic therapy	Fibrosis / cirrhosis regression
16	PMID: 38973543<br>DOI: 10.1097/MEG.0000000000002797	Hepatic encephalopathy therapy; Vasoconstrictor / HRS therapy	Hepatic encephalopathy; Liver function / prognostic scores; Survival / transplant-free outcomes; Ascites / fluid control
17	PMID: 38967589<br>DOI: 10.1097/HC9.0000000000000467	Antifibrotic / metabolic therapy	Fibrosis / cirrhosis regression; Liver function / prognostic scores
18	PMID: 38809154<br>DOI: 10.1097/HEP.0000000000000937	Antifibrotic / metabolic therapy	Renal / HRS outcomes
19	PMID: 38637454<br>DOI: 10.1007/s10620-024-08423-8	Albumin / volume expansion; Vasoconstrictor / HRS therapy	Renal / HRS outcomes; Liver function / prognostic scores; Survival / transplant-free outcomes
20	PMID: 38123994<br>DOI: 10.1136/gutjnl-2023-330419	Portal hypertension / variceal therapy	Portal pressure / variceal bleeding; Liver function / prognostic scores; Survival / transplant-free outcomes; HCC / liver neoplasms
21	PMID: 37942950<br>DOI: 10.14309/ajg.0000000000002575	Hepatic encephalopathy therapy	Hepatic encephalopathy; Survival / transplant-free outcomes
22	PMID: 37929952<br>DOI: 10.14309/ajg.0000000000002569	Antiviral / aetiology-directed therapy; Portal hypertension / variceal therapy	Portal pressure / variceal bleeding; Survival / transplant-free outcomes; HCC / liver neoplasms
23	PMID: 37893503<br>DOI: 10.3390/medicina59101785	Antifibrotic / metabolic therapy	Fibrosis / cirrhosis regression
24	PMID: 37801553<br>DOI: 10.1097/LVT.0000000000000277	Vasoconstrictor / HRS therapy	Renal / HRS outcomes; Liver function / prognostic scores; Survival / transplant-free outcomes
25	PMID: 37544418<br>DOI: 10.1016/j.cgh.2023.07.018	Other therapeutic intervention	HCC / liver neoplasms
26	PMID: 37354045<br>DOI: 10.1111/jgh.16266	Hepatic encephalopathy therapy	Fibrosis / cirrhosis regression; Hepatic encephalopathy; Liver function / prognostic scores
27	PMID: 37088310<br>DOI: 10.1016/j.jhep.2023.04.006	Other therapeutic intervention	Fibrosis / cirrhosis regression; Renal / HRS outcomes; Survival / transplant-free outcomes
28	PMID: 37020315<br>DOI: 10.1186/s13063-023-07261-9	Portal hypertension / variceal therapy; Vasoconstrictor / HRS therapy	Fibrosis / cirrhosis regression; Portal pressure / variceal bleeding; Renal / HRS outcomes; Liver function / prognostic scores; Survival / transplant-free outcomes; Ascites / fluid control
29	PMID: 36963815<br>DOI: 10.1136/gutjnl-2022-328222	Other therapeutic intervention	Liver function / prognostic scores; Survival / transplant-free outcomes
30	PMID: 36940891<br>DOI: 10.1016/j.phrs.2023.106737	Antiviral / aetiology-directed therapy; Traditional / adjunctive medicine	Fibrosis / cirrhosis regression; Liver function / prognostic scores; HCC / liver neoplasms
31	PMID: 36928343<br>DOI: 10.1007/s00535-023-01970-3	Antiviral / aetiology-directed therapy	Virological / aetiological response; Fibrosis / cirrhosis regression; HCC / liver neoplasms

No.	PMID / DOI	Intervention theme(s)	Outcome theme(s)
32	PMID: 36811400<br>DOI: 10.1097/HEP.0000000000000316	Portal hypertension / variceal therapy	Portal pressure / variceal bleeding; Liver function / prognostic scores; Survival / transplant-free outcomes
33	PMID: 36692588<br>DOI: 10.1007/s11739-023-03206-x	Portal hypertension / variceal therapy	Portal pressure / variceal bleeding; Liver function / prognostic scores; Survival / transplant-free outcomes
34	PMID: 36593456<br>DOI: 10.1186/s12876-022-02618-x	Other therapeutic intervention	Renal / HRS outcomes; Liver function / prognostic scores
35	PMID: 36368598<br>DOI: 10.1016/j.jhep.2022.10.027	Antifibrotic / metabolic therapy	Fibrosis / cirrhosis regression
36	PMID: 36152764<br>DOI: 10.1016/j.jhep.2022.09.009	Other therapeutic intervention	Hepatic encephalopathy; Liver function / prognostic scores
37	PMID: 35985545<br>DOI: 10.1016/j.jhep.2022.07.018	Antiviral / aetiology-directed therapy; Traditional / adjunctive medicine	Virological / aetiological response; Fibrosis / cirrhosis regression; Survival / transplant-free outcomes; HCC / liver neoplasms
38	PMID: 35972345<br>DOI: 10.1002/hep4.2065	Other therapeutic intervention	Liver function / prognostic scores; Survival / transplant-free outcomes; Ascites / fluid control
39	PMID: 35842120<br>DOI: 10.1016/j.cgh.2022.05.056	Antiviral / aetiology-directed therapy; Antifibrotic / metabolic therapy	Fibrosis / cirrhosis regression; HCC / liver neoplasms
40	PMID: 34859852<br>DOI: 10.26355/eurrev_202111_27239	Other therapeutic intervention	Liver function / prognostic scores; Survival / transplant-free outcomes; Ascites / fluid control
41	PMID: 34780499<br>DOI: 10.1371/journal.pone.0259338	Other therapeutic intervention	Other clinical outcome
42	PMID: 34687130<br>DOI: 10.1111/liv.15085	Antiviral / aetiology-directed therapy	HCC / liver neoplasms
43	PMID: 34670042<br>DOI: 10.1056/NEJMoa2036205	Antifibrotic / metabolic therapy	Fibrosis / cirrhosis regression
44	PMID: 34606933<br>DOI: 10.1053/j.ajkd.2021.08.016	Vasoconstrictor / HRS therapy	Renal / HRS outcomes; Survival / transplant-free outcomes
45	PMID: 34400208<br>DOI: 10.1016/j.jpeds.2021.08.012	Antifibrotic / metabolic therapy	Fibrosis / cirrhosis regression
46	PMID: 34392656<br>DOI: 10.19852/j.cnki.jtcm.2021.03.015	Antiviral / aetiology-directed therapy	Fibrosis / cirrhosis regression
47	PMID: 34392492<br>DOI: 10.1007/s10620-021-07185-x	Other therapeutic intervention	Fibrosis / cirrhosis regression; Hepatic encephalopathy; Survival / transplant-free outcomes
48	PMID: 34364917<br>DOI: 10.1016/j.jhep.2021.07.033	Other therapeutic intervention	Liver function / prognostic scores; Survival / transplant-free outcomes
49	PMID: 34036519<br>DOI: 10.1007/s12072-021-10164-z	Portal hypertension / variceal therapy	Fibrosis / cirrhosis regression; Portal pressure / variceal bleeding; Renal / HRS outcomes; Liver function / prognostic scores; Survival / transplant-free outcomes
50	PMID: 33977364<br>DOI: 10.1007/s12072-021-10188-5	Portal hypertension / variceal therapy	Hepatic encephalopathy; Liver function / prognostic scores
51	PMID: 33908180<br>DOI: 10.1002/jhbp.972	Antifibrotic / metabolic therapy	Fibrosis / cirrhosis regression; Liver function / prognostic scores
52	PMID: 33677771<br>DOI: 10.1007/s12072-021-10162-1	Antiviral / aetiology-directed therapy	Virological / aetiological response; Fibrosis / cirrhosis regression
53	PMID: 33657294<br>DOI: 10.1056/NEJMoa2008290	Vasoconstrictor / HRS therapy	Fibrosis / cirrhosis regression; Renal / HRS outcomes; Liver function / prognostic scores; Survival / transplant-free outcomes

No.	PMID / DOI	Intervention theme(s)	Outcome theme(s)
54	PMID: 33169409<br>DOI: 10.1002/hep.31622	Antifibrotic / metabolic therapy	Fibrosis / cirrhosis regression; Liver function / prognostic scores
55	PMID: 32990234<br>DOI: 10.12122/j.issn.1673-4254.2020.09.07	Other therapeutic intervention	Other clinical outcome
56	PMID: 32853747<br>DOI: 10.1016/j.jhep.2020.08.021	Albumin / volume expansion	Liver function / prognostic scores; Survival / transplant-free outcomes; Ascites / fluid control
57	PMID: 32437567<br>DOI: 10.1093/infdis/jiaa266	Antiviral / aetiology-directed therapy; Antifibrotic / metabolic therapy; Traditional / adjunctive medicine	Fibrosis / cirrhosis regression
58	PMID: 32048131<br>DOI: 10.1007/s12072-020-10013-5	Portal hypertension / variceal therapy	Portal pressure / variceal bleeding; Renal / HRS outcomes; Liver function / prognostic scores; Survival / transplant-free outcomes
59	PMID: 31987796<br>DOI: 10.1053/j.gastro.2020.01.031	Hepatic encephalopathy therapy; Antifibrotic / metabolic therapy	Other clinical outcome
60	PMID: 31528100<br>DOI: 10.3748/wjg.v25.i32.4764	Antiviral / aetiology-directed therapy	Virological / aetiological response; Fibrosis / cirrhosis regression
61	PMID: 31448533<br>DOI: 10.1111/1751-2980.12816	Hepatic encephalopathy therapy	Hepatic encephalopathy
62	PMID: 31390762<br>DOI: 10.3390/nu11081810	Hepatic encephalopathy therapy	Hepatic encephalopathy
63	PMID: 31357778<br>DOI: 10.3760/cma.j.issn.1007-3418.2019.07.009	Antiviral / aetiology-directed therapy	Fibrosis / cirrhosis regression
64	PMID: 30905065<br>DOI: 10.1002/jmv.25465	Antiviral / aetiology-directed therapy	Fibrosis / cirrhosis regression; Liver function / prognostic scores
65	PMID: 30887050<br>DOI: 10.1093/ndt/gfz048	Vasoconstrictor / HRS therapy	Renal / HRS outcomes; Liver function / prognostic scores; Survival / transplant-free outcomes
66	PMID: 30222962<br>DOI: 10.1053/j.gastro.2018.09.021	Antifibrotic / metabolic therapy	Liver function / prognostic scores; Survival / transplant-free outcomes
67	PMID: 30178092<br>DOI: 10.1007/s12664-018-0876-3	Vasoconstrictor / HRS therapy	Fibrosis / cirrhosis regression; Renal / HRS outcomes; Liver function / prognostic scores; Survival / transplant-free outcomes
68	PMID: 30076614<br>DOI: 10.1002/hep.30208	Vasoconstrictor / HRS therapy	Renal / HRS outcomes; Liver function / prognostic scores; Survival / transplant-free outcomes
69	PMID: 29893462<br>DOI: 10.1111/liv.13900	Antiviral / aetiology-directed therapy	Virological / aetiological response
70	PMID: 29080299<br>DOI: 10.1111/jvh.12814	Antiviral / aetiology-directed therapy	Fibrosis / cirrhosis regression; Liver function / prognostic scores
71	PMID: 28827788<br>DOI: 10.1038/s41598-017-09512-7	Other therapeutic intervention	Other clinical outcome
72	PMID: 28749571<br>DOI: 10.1002/hep.29410	Hepatic encephalopathy therapy	Fibrosis / cirrhosis regression; Hepatic encephalopathy; Renal / HRS outcomes
73	PMID: 28245243<br>DOI: 10.1371/journal.pmed.1002248	Portal hypertension / variceal therapy; Vasoconstrictor / HRS therapy	Fibrosis / cirrhosis regression; Portal pressure / variceal bleeding; Renal / HRS outcomes
74	PMID: 28051800<br>DOI: 10.5604/16652681.1226822	Hepatic encephalopathy therapy	Hepatic encephalopathy; Liver function / prognostic scores; Survival / transplant-free outcomes
75	PMID: 27906753<br>DOI: 10.1097/MEG.0000000000000789	Antiviral / aetiology-directed therapy; Antifibrotic / metabolic therapy	Fibrosis / cirrhosis regression
76	PMID: 27762512	Vasoconstrictor / HRS therapy	Renal / HRS outcomes; Liver function / prognostic scores

No.	PMID / DOI	Intervention theme(s)	Outcome theme(s)
77	PMID: 27565061<br>DOI: 10.1016/j.jss.2016.04.036	Portal hypertension / variceal therapy	Portal pressure / variceal bleeding; HCC / liver neoplasms
78	PMID: 27439433<br>DOI: 10.1186/s13063-016-1454-6	Antiviral / aetiology-directed therapy; Antifibrotic / metabolic therapy	Virological / aetiological response; Fibrosis / cirrhosis regression; HCC / liver neoplasms
79	PMID: 27084721<br>DOI: 10.1007/s12325-016-0320-y	Antiviral / aetiology-directed therapy	Liver function / prognostic scores
80	PMID: 27064832<br>DOI: 10.1089/lap.2016.0012	Portal hypertension / variceal therapy	Portal pressure / variceal bleeding
81	PMID: 27022224<br>DOI: 10.3748/wjg.v22.i12.3418	Antiviral / aetiology-directed therapy	Virological / aetiological response
82	PMID: 27009831<br>DOI: 10.1111/liv.13128	Antiviral / aetiology-directed therapy	Virological / aetiological response; Liver function / prognostic scores
83	PMID: 26951571<br>DOI: 10.1093/cid/ciw126	Other therapeutic intervention	Other clinical outcome
84	PMID: 26896734<br>DOI: 10.1053/j.gastro.2016.02.026	Vasoconstrictor / HRS therapy	Renal / HRS outcomes; Liver function / prognostic scores; Survival / transplant-free outcomes; Ascites / fluid control
85	PMID: 26874076<br>DOI: 10.1053/j.gastro.2016.01.038	Antifibrotic / metabolic therapy	Renal / HRS outcomes
86	PMID: 26707685<br>DOI: 10.1016/j.cgh.2015.11.018	Portal hypertension / variceal therapy	Portal pressure / variceal bleeding; Hepatic encephalopathy
87	PMID: 26653681<br>DOI: 10.1148/radiol.2015150369	Portal hypertension / variceal therapy	Portal pressure / variceal bleeding; Hepatic encephalopathy; Survival / transplant-free outcomes
88	PMID: 26599983<br>DOI: 10.1002/hep.28352	Portal hypertension / variceal therapy	Portal pressure / variceal bleeding; Renal / HRS outcomes; Liver function / prognostic scores; Survival / transplant-free outcomes; Ascites / fluid control
89	PMID: 26573188<br>DOI: 10.3760/cma.j.issn.1007-3418.2015.10.005	Antiviral / aetiology-directed therapy	Virological / aetiological response; Renal / HRS outcomes; Liver function / prognostic scores; Survival / transplant-free outcomes; HCC / liver neoplasms
90	PMID: 26453952<br>DOI: 10.1016/j.cgh.2015.09.028	Portal hypertension / variceal therapy; Hepatic encephalopathy therapy	Portal pressure / variceal bleeding; Hepatic encephalopathy
91	PMID: 26206073<br>DOI: 10.1016/j.jhep.2015.07.007	Hepatic encephalopathy therapy	Hepatic encephalopathy; Renal / HRS outcomes; Liver function / prognostic scores; Ascites / fluid control
92	PMID: 26201713<br>DOI: 10.1111/liv.12921	Hepatic encephalopathy therapy	Hepatic encephalopathy
93	PMID: 26078579<br>DOI: 10.3748/wjg.v21.i22.7008	Antifibrotic / metabolic therapy	Fibrosis / cirrhosis regression; Liver function / prognostic scores
94	PMID: 25788199<br>DOI: 10.1007/s12072-015-9614-4	Antiviral / aetiology-directed therapy	Virological / aetiological response; Fibrosis / cirrhosis regression; Liver function / prognostic scores; Survival / transplant-free outcomes; HCC / liver neoplasms
95	PMID: 25644760<br>DOI: 10.1002/hep.27709	Vasoconstrictor / HRS therapy	Renal / HRS outcomes; Liver function / prognostic scores; Survival / transplant-free outcomes
96	PMID: 25613115<br>DOI: 10.1158/1055-9965.EPI-14-1038	Other therapeutic intervention	Liver function / prognostic scores; Survival / transplant-free outcomes; HCC / liver neoplasms
97	PMID: 25532501<br>DOI: 10.1007/s10620-014-3486-7	Antiviral / aetiology-directed therapy	Virological / aetiological response; Renal / HRS outcomes; Liver function / prognostic scores

No.	PMID / DOI	Intervention theme(s)	Outcome theme(s)
98	PMID: 25526495<br>DOI: 10.1097/MD.0000000000000338	Other therapeutic intervention	Renal / HRS outcomes; Liver function / prognostic scores; Survival / transplant-free outcomes
99	PMID: 25469044<br>DOI: 10.3748/wjg.v20.i44.16726	Antiviral / aetiology-directed therapy	Virological / aetiological response
100	PMID: 25381721<br>DOI: 10.1186/1745-6215-15-438	Antiviral / aetiology-directed therapy; Traditional / adjunctive medicine	Fibrosis / cirrhosis regression; Survival / transplant-free outcomes; HCC / liver neoplasms
101	PMID: 25369449<br>DOI: 10.1148/radiol.14140933	Antifibrotic / metabolic therapy	Other clinical outcome
102	PMID: 25179493<br>DOI: 10.1007/s10620-014-3336-7	Antiviral / aetiology-directed therapy	Virological / aetiological response; Fibrosis / cirrhosis regression; Renal / HRS outcomes
103	PMID: 25135867<br>DOI: 10.1016/j.jhep.2014.08.013	Other therapeutic intervention	Fibrosis / cirrhosis regression
104	PMID: 25043514<br>DOI: 10.1002/hep.27289	Antifibrotic / metabolic therapy	Other clinical outcome
105	PMID: 25038208<br>DOI: 10.4103/1319-3767.136975	Hepatic encephalopathy therapy	Fibrosis / cirrhosis regression; Hepatic encephalopathy
106	PMID: 23877348<br>DOI: 10.1038/ajg.2013.219	Hepatic encephalopathy therapy	Hepatic encephalopathy; Renal / HRS outcomes; Liver function / prognostic scores; Survival / transplant-free outcomes
107	PMID: 23800784<br>DOI: 10.1016/j.jinf.2013.05.007	Antiviral / aetiology-directed therapy	Fibrosis / cirrhosis regression; Survival / transplant-free outcomes
108	PMID: 23750991<br>DOI: 10.1111/apt.12370	Other therapeutic intervention	Survival / transplant-free outcomes; HCC / liver neoplasms
109	PMID: 23485720<br>DOI: 10.1159/000346083	Hepatic encephalopathy therapy	Hepatic encephalopathy; Liver function / prognostic scores
110	PMID: 23258530<br>DOI: 10.1002/hep.26203	Antiviral / aetiology-directed therapy	Virological / aetiological response; Fibrosis / cirrhosis regression; Survival / transplant-free outcomes; HCC / liver neoplasms
111	PMID: 23234725<br>DOI: 10.1016/S0140-6736(12)61425-1	Antiviral / aetiology-directed therapy	Virological / aetiological response; Fibrosis / cirrhosis regression
112	PMID: 22619107<br>DOI: 10.1002/hep.25867	Antiviral / aetiology-directed therapy	Virological / aetiological response; Fibrosis / cirrhosis regression
113	PMID: 22613001<br>DOI: 10.1016/j.jhep.2012.04.022	Antiviral / aetiology-directed therapy	Virological / aetiological response; Fibrosis / cirrhosis regression; Renal / HRS outcomes; Survival / transplant-free outcomes
114	PMID: 21756847<br>DOI: 10.1016/j.jhep.2011.05.022	Antiviral / aetiology-directed therapy	Virological / aetiological response; Fibrosis / cirrhosis regression
115	PMID: 21699796<br>DOI: 10.1053/j.gastro.2011.06.007	Antiviral / aetiology-directed therapy	Fibrosis / cirrhosis regression; Survival / transplant-free outcomes; HCC / liver neoplasms
116	PMID: 21640198<br>DOI: 10.1016/j.cgh.2011.04.029	Antiviral / aetiology-directed therapy	Virological / aetiological response; Liver function / prognostic scores
117	PMID: 21440548<br>DOI: 10.1053/j.gastro.2011.03.045	Antiviral / aetiology-directed therapy	HCC / liver neoplasms
118	PMID: 21410752<br>DOI: 10.1111/j.1440-1746.2011.06722.x	Antiviral / aetiology-directed therapy; Antifibrotic / metabolic therapy	Fibrosis / cirrhosis regression
119	PMID: 21207172<br>DOI: 10.1245/s10434-010-1458-5	Other therapeutic intervention	HCC / liver neoplasms
120	PMID: 21157444<br>DOI: 10.1038/ajg.2010.455	Hepatic encephalopathy therapy	Hepatic encephalopathy

No.	PMID / DOI	Intervention theme(s)	Outcome theme(s)
121	PMID: 21059778<br>DOI: 10.1503/cmaj.090707	Other therapeutic intervention	Survival / transplant-free outcomes
122	PMID: 20352147<br>DOI: 10.1155/2010/349076	Other therapeutic intervention	Survival / transplant-free outcomes
123	PMID: 19766645<br>DOI: 10.1053/j.gastro.2009.08.071	Antiviral / aetiology-directed therapy	Virological / aetiological response
124	PMID: 19500693<br>DOI: 10.1016/j.cgh.2009.05.025	Portal hypertension / variceal therapy	Virological / aetiological response; Portal pressure / variceal bleeding; Survival / transplant-free outcomes; HCC / liver neoplasms; Ascites / fluid control
125	PMID: 19486469<br>DOI: 10.1111/j.1365-2893.2009.01122.x	Antiviral / aetiology-directed therapy	Virological / aetiological response
126	PMID: 19233692<br>DOI: 10.1016/j.bcmd.2009.01.002	Other therapeutic intervention	Liver function / prognostic scores; Survival / transplant-free outcomes
127	PMID: 18471513<br>DOI: 10.1053/j.gastro.2008.02.014	Vasoconstrictor / HRS therapy	Renal / HRS outcomes; Liver function / prognostic scores; Survival / transplant-free outcomes
128	PMID: 18458640<br>DOI: 10.1097/MCG.0b013e31815cff36	Other therapeutic intervention	Fibrosis / cirrhosis regression
129	PMID: 18250126<br>DOI: 10.1136/gut.2007.137489	Portal hypertension / variceal therapy	Liver function / prognostic scores; Survival / transplant-free outcomes
130	PMID: 18208570<br>DOI: 10.1111/j.1365-2036.2008.03620.x	Antiviral / aetiology-directed therapy	Virological / aetiological response
131	PMID: 17687341<br>DOI: 10.1038/sj.bjc.6603901	Portal hypertension / variceal therapy; Vasoconstrictor / HRS therapy	Portal pressure / variceal bleeding; Liver function / prognostic scores; Survival / transplant-free outcomes; HCC / liver neoplasms
132	PMID: 17419259	Portal hypertension / variceal therapy; Vasoconstrictor / HRS therapy	Portal pressure / variceal bleeding; Liver function / prognostic scores; Survival / transplant-free outcomes
133	PMID: 17327945<br>DOI: 10.1080/00365520600955526	Antiviral / aetiology-directed therapy	Virological / aetiological response; Liver function / prognostic scores
134	PMID: 17162245<br>DOI: 10.1016/j.cgh.2006.09.025	Antifibrotic / metabolic therapy	Liver function / prognostic scores
135	PMID: 17140886<br>DOI: 10.1016/j.gie.2006.03.007	Antiviral / aetiology-directed therapy; Portal hypertension / variceal therapy	Portal pressure / variceal bleeding; Liver function / prognostic scores
136	PMID: 17133499<br>DOI: 10.1002/hep.21440	Antiviral / aetiology-directed therapy	Virological / aetiological response; Fibrosis / cirrhosis regression
137	PMID: 17133492<br>DOI: 10.1002/hep.21415	Antiviral / aetiology-directed therapy	Virological / aetiological response; Survival / transplant-free outcomes; HCC / liver neoplasms
138	PMID: 16789980<br>DOI: 10.1111/j.1365-2796.2006.01654.x	Vasoconstrictor / HRS therapy	Liver function / prognostic scores; Ascites / fluid control
139	PMID: 16557539<br>DOI: 10.1002/hep.21145	Portal hypertension / variceal therapy	Portal pressure / variceal bleeding; Survival / transplant-free outcomes; HCC / liver neoplasms
140	PMID: 16518754<br>DOI: 10.1086/500952	Antiviral / aetiology-directed therapy	Virological / aetiological response; Fibrosis / cirrhosis regression
141	PMID: 16234012<br>DOI: 10.1016/s1542-3565(05)00404-0	Antiviral / aetiology-directed therapy; Antifibrotic / metabolic therapy	Virological / aetiological response

No.	PMID / DOI	Intervention theme(s)	Outcome theme(s)
142	PMID: 15973099<br>DOI: 10.1097/01.sla.0000167925.90380.fe	Other therapeutic intervention	Survival / transplant-free outcomes; HCC / liver neoplasms; Ascites / fluid control
143	PMID: 15854180<br>DOI: 10.1111/j.1365-2036.2005.02453.x	Antiviral / aetiology-directed therapy	Other clinical outcome
144	PMID: 15329205	Antifibrotic / metabolic therapy; Traditional / adjunctive medicine	Fibrosis / cirrhosis regression
145	PMID: 15316334<br>DOI: 10.1097/00002030-200409030-00002	Antiviral / aetiology-directed therapy	Liver function / prognostic scores
146	PMID: 15188169<br>DOI: 10.1053/j.gastro.2004.03.017	Antiviral / aetiology-directed therapy	Virological / aetiological response; Fibrosis / cirrhosis regression; Liver function / prognostic scores; Survival / transplant-free outcomes
147	PMID: 15122774<br>DOI: 10.1002/hep.20194	Hepatic encephalopathy therapy	Fibrosis / cirrhosis regression; Hepatic encephalopathy
148	PMID: 14678008<br>DOI: 10.1042/CS20030357	Albumin / volume expansion	Fibrosis / cirrhosis regression; Hepatic encephalopathy; Renal / HRS outcomes; Liver function / prognostic scores; Ascites / fluid control
149	PMID: 12759473<br>DOI: 10.1148/radiol.2281020718	Other therapeutic intervention	Liver function / prognostic scores; Survival / transplant-free outcomes; HCC / liver neoplasms
150	PMID: 12480560<br>DOI: 10.1016/s0168-8278(02)00350-1	Hepatic encephalopathy therapy	Hepatic encephalopathy
151	PMID: 12217604<br>DOI: 10.1016/s0168-8278(02)00211-8	Antiviral / aetiology-directed therapy	Virological / aetiological response; Liver function / prognostic scores
152	PMID: 12174378<br>DOI: 10.3748/wjg.v8.i4.679	Antiviral / aetiology-directed therapy; Traditional / adjunctive medicine	Fibrosis / cirrhosis regression; Liver function / prognostic scores
153	PMID: 12063979	Antiviral / aetiology-directed therapy	Liver function / prognostic scores; Survival / transplant-free outcomes; HCC / liver neoplasms
154	PMID: 11547718<br>DOI: 10.1056/NEJMoa003223	Portal hypertension / variceal therapy	Portal pressure / variceal bleeding; Liver function / prognostic scores; Survival / transplant-free outcomes
155	PMID: 11093724<br>DOI: 10.1053/jhep.2000.20240	Other therapeutic intervention	Other clinical outcome
156	PMID: 10607223<br>DOI: 10.1046/j.1365-2893.1999.00148.x	Antiviral / aetiology-directed therapy	Fibrosis / cirrhosis regression
157	PMID: 10419923<br>DOI: 10.1053/gast.1999.0029900408	Antiviral / aetiology-directed therapy	Virological / aetiological response
158	PMID: 10419922<br>DOI: 10.1053/gast.1999.0029900400	Other therapeutic intervention	Fibrosis / cirrhosis regression; Liver function / prognostic scores; Survival / transplant-free outcomes
159	PMID: 9581697<br>DOI: 10.1002/hep.510270529	Antiviral / aetiology-directed therapy	Virological / aetiological response; HCC / liver neoplasms; Survival / transplant-free outcomes
160	PMID: 9566819<br>DOI: 10.1016/s0168-8278(98)80274-2	Antiviral / aetiology-directed therapy	Virological / aetiological response; Liver function / prognostic scores; Survival / transplant-free outcomes; HCC / liver neoplasms
161	PMID: 9007710<br>DOI: 10.1016/s0168-8278(96)80286-8	Antiviral / aetiology-directed therapy	Virological / aetiological response; Fibrosis / cirrhosis regression
162	PMID: 8907566<br>DOI: 10.1016/s0168-8278(96)80022-5	Antiviral / aetiology-directed therapy	HCC / liver neoplasms

No.	PMID / DOI	Intervention theme(s)	Outcome theme(s)
163	PMID: 8550048<br>DOI: 10.1002/hep.510230108	Other therapeutic intervention	Fibrosis / cirrhosis regression
164	PMID: 7884615<br>DOI: 10.1097/00005176-199501000-00006	Other therapeutic intervention	Fibrosis / cirrhosis regression; Survival / transplant-free outcomes
165	PMID: 8152446<br>DOI: 10.1056/NEJM199405123301903	Other therapeutic intervention	Liver function / prognostic scores; Portal pressure / variceal bleeding; Hepatic encephalopathy; Ascites / fluid control; Survival / transplant-free outcomes
166	PMID: 8244259	Antiviral / aetiology-directed therapy	Fibrosis / cirrhosis regression
167	PMID: 1674105<br>DOI: 10.1056/NEJM199105303242204	Other therapeutic intervention	Liver function / prognostic scores; Portal pressure / variceal bleeding; Ascites / fluid control
168	PMID: 3899841<br>DOI: 10.1016/0016-5085(85)90213-6	Other therapeutic intervention	Liver function / prognostic scores; Survival / transplant-free outcomes

Note. The list contains 168 PubMed RCT publications retrieved from database inception to May 31, 2026. Intervention and outcome themes were assigned according to the operational definitions and matching framework provided in Supplementary Table 8. Matching was applied non-exclusively to titles, abstracts, and available keyword fields, followed by record-level review of ambiguous assignments. A publication could therefore be assigned to more than one intervention or outcome theme. Formal Baveno VII recompensation was prespecified as an outcome category, but no publication met its operational definition. HCC, hepatocellular carcinoma; HRS, hepatorenal syndrome; RCT, randomized controlled trial.

2 Supplementary Figures

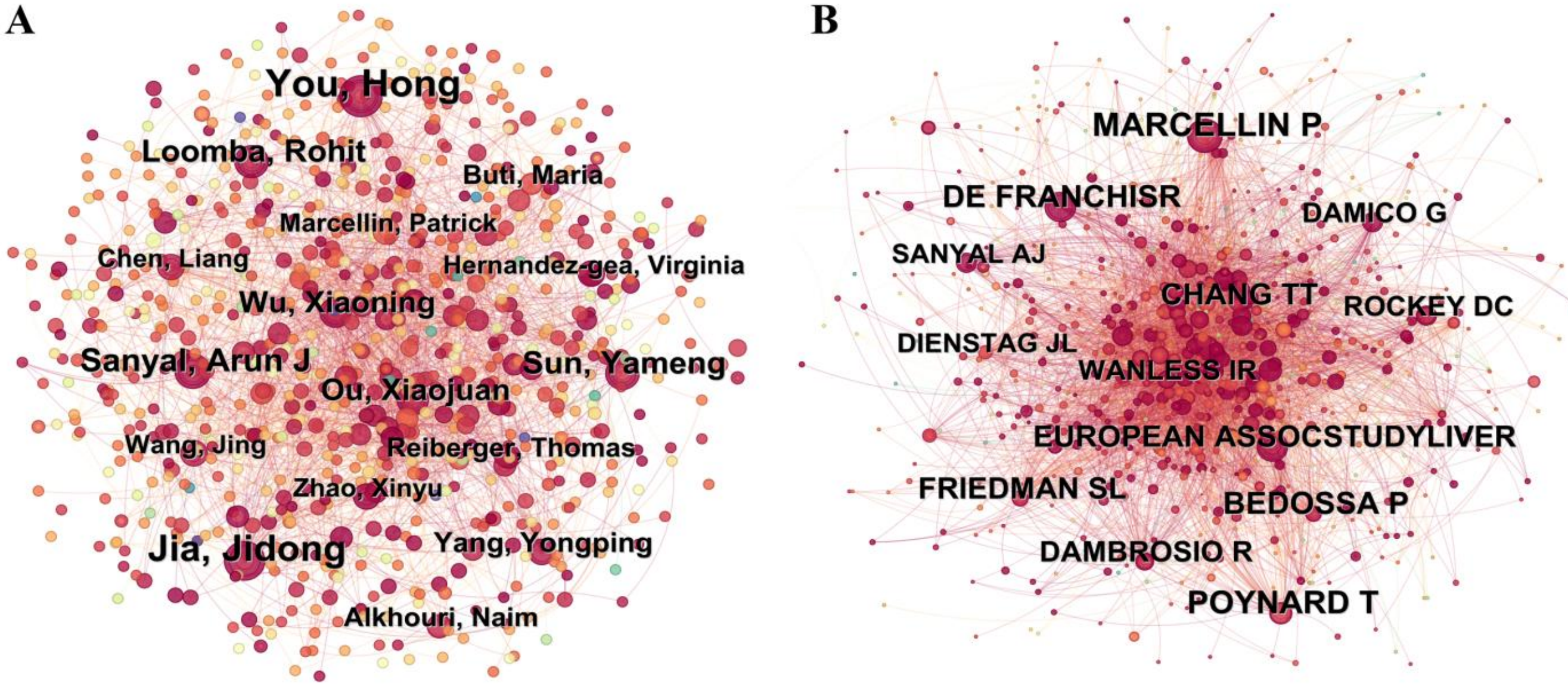

Supplementary Figure 1. Author collaboration and co-cited author networks in cirrhosis disease-modification and recompensation-related research. (A) Author collaboration network. (B) Co-cited author network. Node size reflects publication or citation frequency, and links indicate collaboration or co-citation relationships.

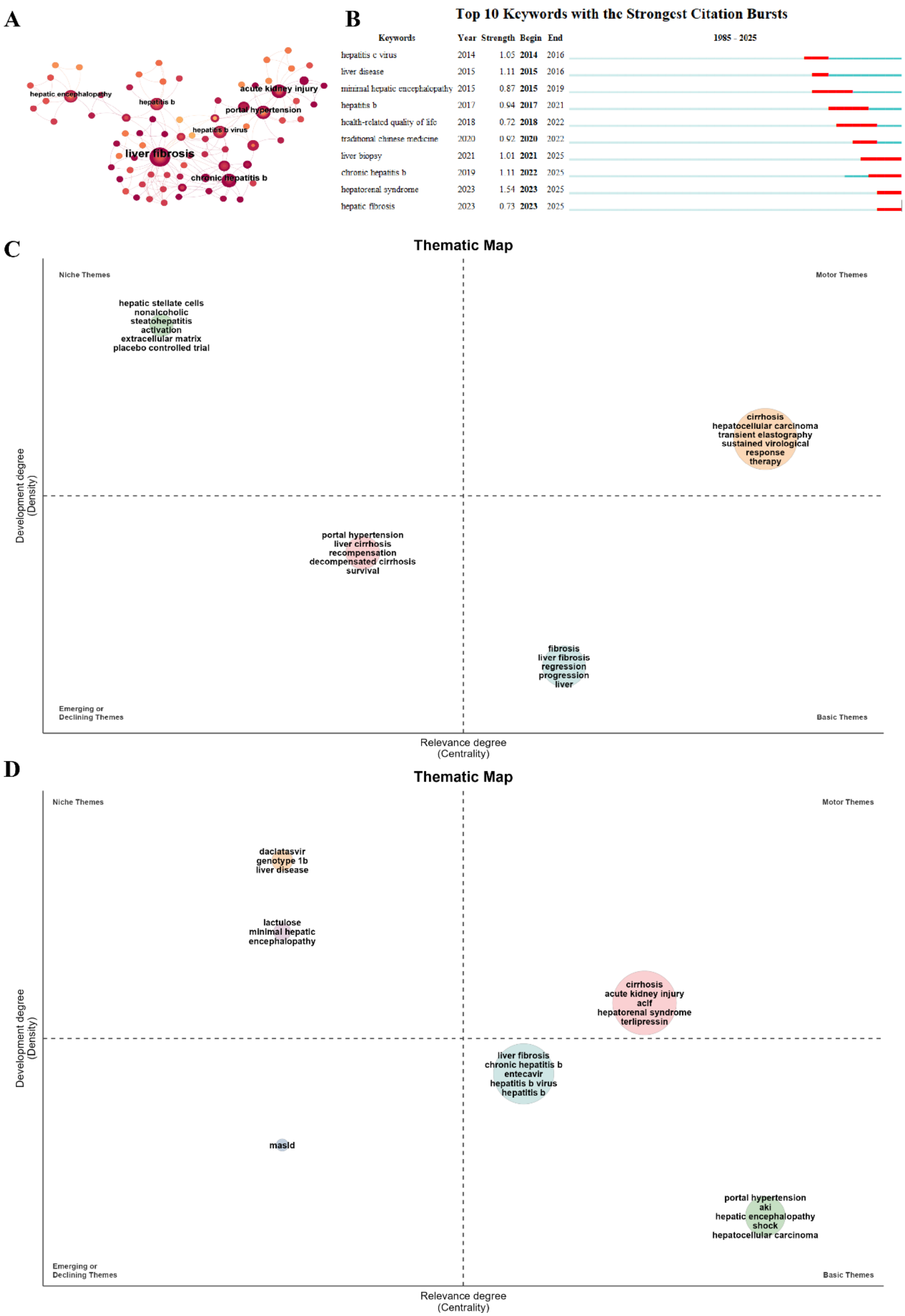

Supplementary Figure 2. PubMed RCT evidence mapping and separately generated thematic maps. (A) Keyword co-occurrence network of the 168 PubMed RCT publications. Node size is proportional to keyword occurrence frequency, links indicate co-occurrence within the same publication, and link width

represents co-occurrence strength. Node and link colors indicate the time slices in which keywords and connections appeared. The network was generated in CiteSpace 7.0 using one-year time slices, the g-index with k = 25, Pathfinder, pruning sliced networks, and pruning the merged network. (B) Top 10 keywords with the strongest citation bursts during 1985–2025. The light-blue line represents the complete observation period, and red segments indicate the active burst intervals; strength denotes the burst intensity, while begin and end indicate the corresponding years. (C, D) Thematic maps of the 588-publication WoSCC dataset and the 168-publication PubMed RCT dataset, respectively. Themes were positioned according to centrality on the x-axis and density on the y-axis, with dashed lines indicating the median values used to define motor, niche, emerging or declining, and basic themes. Bubble size is proportional to the cumulative frequency of keywords assigned to each theme. Keyword and theme assignments were non-exclusive; therefore, one publication could contribute to more than one theme.

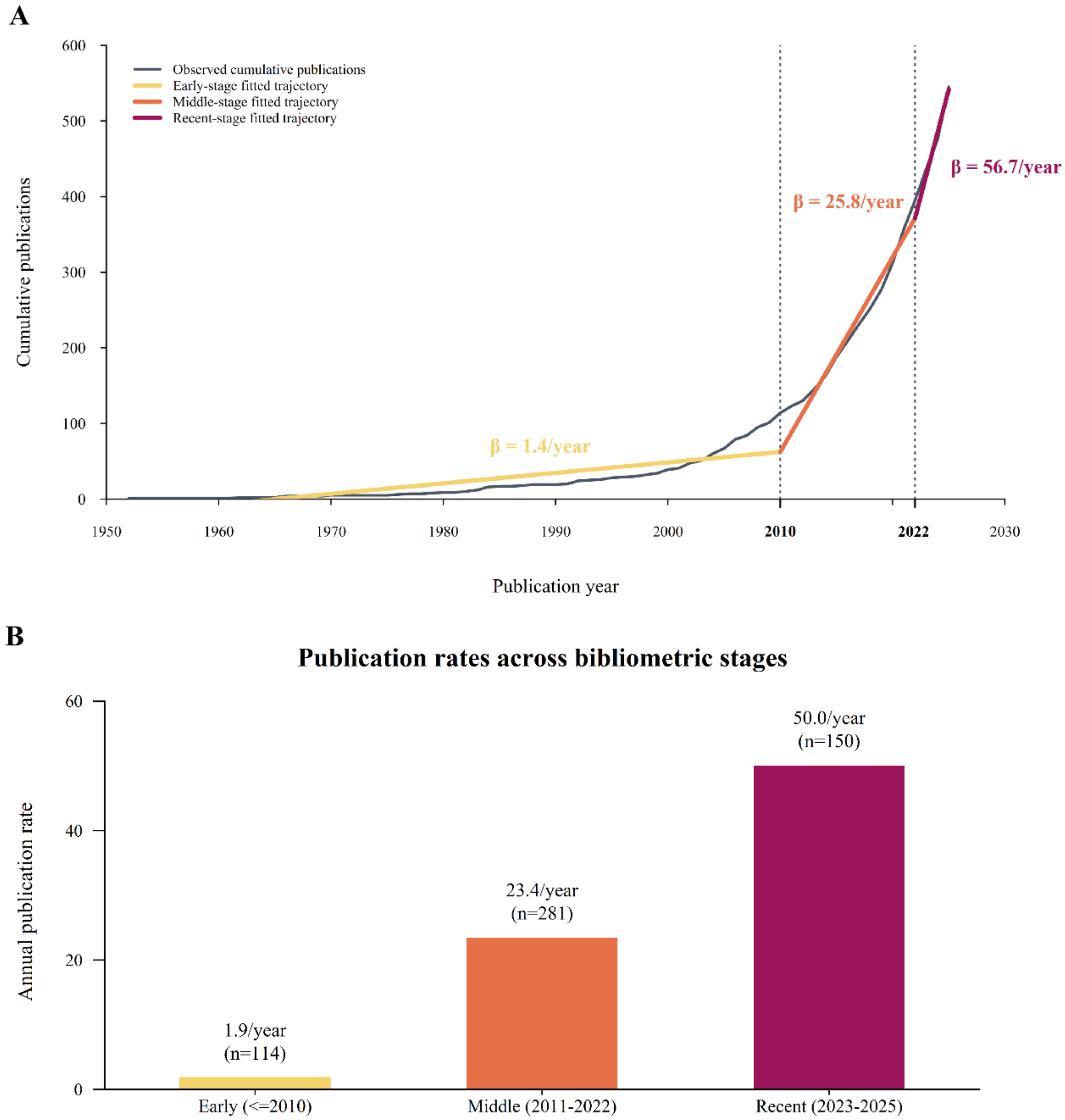

Supplementary Figure 3. Quantitative assessment of publication dynamics across the three pre-specified bibliometric stages. (A) Observed cumulative publication counts and piecewise linear fitted trajectories with fixed knots at 2010 and 2022. The dotted vertical lines indicate the two pre-specified transition years. β represents the fitted annual increase in cumulative publications within each stage, increasing from 1.4 publications/year in the early stage to 25.8 publications/year in the middle stage and 56.7 publications/year in the recent stage. (B) Observed annual publication rates across the early (≤2010; 114 publications, 1.9/year), middle (2011–2022; 281 publications, 23.4/year), and recent stages (2023–2025; 150 publications, 50.0/year). The incomplete year 2026 was excluded from the rate calculations. Poisson rate tests showed a 12.12-fold increase from the early to middle stage (95% CI, 9.75–15.07; P<0.001) and a 2.14-fold increase from the middle to recent stage (95% CI, 1.75–2.60; P<0.001; Supplementary Table 2). These results provide quantitative support for the 2010 and 2022 transition points and the proposed three-stage framework, independently of the citation-burst evidence. Panel A reports model-estimated slopes from a continuous piecewise linear regression with fixed knots, whereas Panel B reports observed stage-specific publication

counts divided by the number of complete years. Therefore, the fitted slopes in Panel A are not expected to equal the observed mean annual publication rates in Panel B.

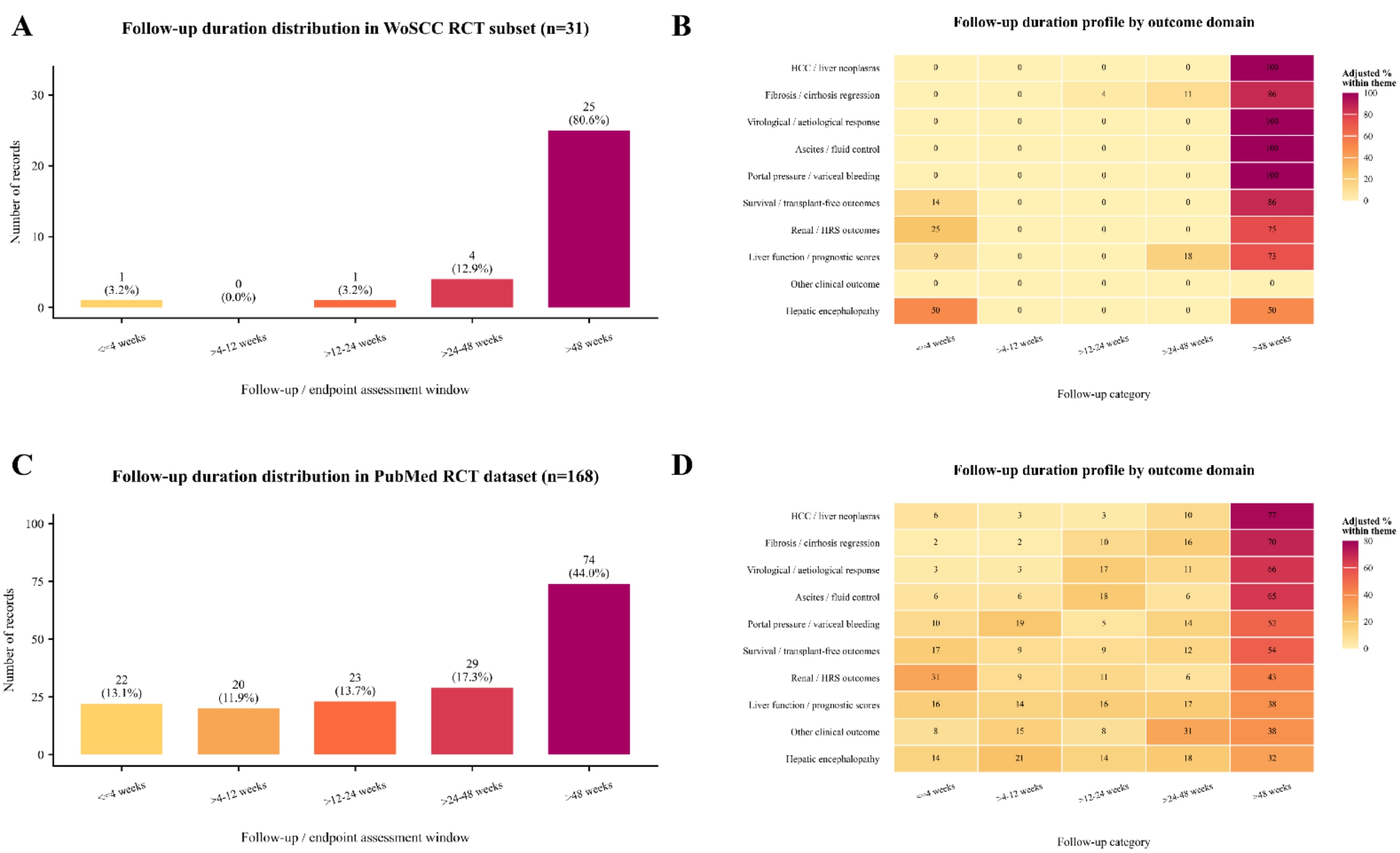

Supplementary Figure 4. Follow-up duration profiles in the WoSCC RCT subset and complementary PubMed RCT dataset. (A) Follow-up distribution in the 31-publication WoSCC RCT subset, with 25 publications (80.6%) assigned to >48 weeks. (B) Follow-up profiles across outcome domains in the WoSCC subset. (C) Follow-up distribution in the 168-publication PubMed RCT dataset, with 74 publications (44.0%) assigned to >48 weeks. (D) No publication used formal Baveno VII recompensation as an explicit endpoint, including those with follow-up beyond 48 weeks, indicating that this gap cannot be explained by short follow-up alone.
